# *Bacillus subtilis* 26D Triggers Induced Systemic Resistance against *Rhopalosiphum padi* L. by Regulating the Expression of Genes *AGO*, *DCL* and microRNA in Bread Spring Wheat

**DOI:** 10.3390/microorganisms11122983

**Published:** 2023-12-14

**Authors:** Sergey D. Rumyantsev, Svetlana V. Veselova, Guzel F. Burkhanova, Valentin Y. Alekseev, Igor V. Maksimov

**Affiliations:** Institute of Biochemistry and Genetics, Ufa Federal Research Centre, Russian Academy of Sciences, Prospekt Oktyabrya, 71, 450054 Ufa, Russia; rumyantsev-serg@mail.ru (S.D.R.); guzel_mur@mail.ru (G.F.B.); valentin-1994@yandexx.ru (V.Y.A.); igor.mak2011@yandex.ru (I.V.M.)

**Keywords:** *Bacillus subtilis*, *Rhopalosiphum padi*, endophytes, phloem-feeding insects, phytohormones, induced systemic resistance (ISR), RNA interference, *AGO*, *DCL*, microRNAs

## Abstract

*Bacillus subtilis* 26D is a plant growth-promoting endophytic bacteria capable of inducing systemic resistance through the priming mechanism, which includes plant genome reprogramming and the phenomenon of RNA interference (RNAi) and microRNA (miRNAs). The phloem-feeding insect bird cherry-oat aphid *Rhopalosiphum padi* L. is a serious pest that causes significant damage to crops throughout the world. However, the function of plant miRNAs in the response to aphid infestation remains unclear. The results of this work showed that *B. subtilis* 26D stimulated aphid resistance in wheat plants, inducing the expression of genes of hormonal signaling pathways *ICS*, *WRKY13*, *PR1*, *ACS*, *EIN3*, *PR3,* and *ABI5*. In addition, *B. subtilis* 26D activated the RNAi mechanism and regulated the expression of nine conserved miRNAs through activation of the ethylene, salicylic acid (SA), and abscisic acid (ABA) signaling pathways, which was demonstrated by using treatments with phytohormones. Treatment of plants with SA, ethylene, and ABA acted in a similar manner to *B. subtilis* 26D on induction of the expression of the *AGO4*, *AGO5* and *DCL2*, *DCL4* genes, as well as the expression of nine conserved miRNAs. Different patterns of miRNA expression were found in aphid-infested plants and in plants treated with *B. subtilis* 26D or SA, ethylene, and ABA and infested by aphids, suggesting that miRNAs play multiple roles in the plant response to phloem-feeding insects, associated with effects on hormonal signaling pathways, redox metabolism, and the synthesis of secondary metabolites. Our study provides new data to further elucidate the fine mechanisms of bacterial-induced priming. However, further extensive work is needed to fully unravel these mechanisms.

## 1. Introduction

Insect pests that feed on phloem sap (aphids, whiteflies, and leafhoppers) cause significant damage to agricultural crops. Bird cherry-oat aphid *Rhopalosiphum padi* L. is a migratory aphid species and is widespread throughout the world [1,2]. *R. padi* significantly reduces wheat yield, as aphids uptake phloem sap during feeding and reduce the rate of photosynthesis in plants [3,4]. Unfortunately, few biocontrol agents are currently available against sap-sucking insects, and control of these pests is limited to the use of chemical systemic insecticides that pollute the environment. Therefore, plant growth-promoting bacteria (PGPB), which induce immune responses in plants, are of great interest in agriculture [5,6].

Recently, studies have appeared that prove the development of defensive reactions in plants under the influence of PGPB during an insect attack [7,8,9]. However, most research works and reviews are devoted to studying the influence of bacteria on the development of protective reactions in plants against insects with chewing oral apparatus [7,8,10]. There is much less work on the PGPB effect on the immune reactions of plants against phloem-feeding insects [9,11,12].

One of the PGPB-mediated mechanisms of plant defense is associated with the triggering of induced systemic resistance (ISR) in plants. PGPB-mediated ISR occurs via microbe-associated molecular patterns (MAMPs), such as flagellin, lipopolysaccharides, siderophores, antibiotics, and biosurfactants, as well as volatile organic compounds, and is regulated by phytohormones-jasmonic (JA) and salicylic acids (SA), ethylene, abscisic acid (ABA), as well as cytokinins (CK) and auxins [5,6,13,14]. It has been shown that bacteria trigger ISR in plants against sap-sucking insects via the ethylene/JA and SA defensive pathways [11,12,15,16].

The distinctive feature of PGPB-mediated ISR is the development of resistance through the priming mechanism [14,17,18]. Bacteria-mediated ISR developing along the priming pathway is expressed in the host genome reprogramming at various stages of interaction with harmful organisms. Early responses are characterized by the rapid accumulation of reactive oxygen species (ROS), which activate redox-sensitive transcription factors (TF) from various families (ERF, MYB, MYC, WRKY) and pathogenesis-related proteins (PR-proteins) genes [13,14]. Long-term responses involve epigenetic regulation of plant gene expression, including DNA (de)methylation and RNA-directed DNA methylation (RdDM) with the participation of plant small RNAs (sRNAs) [18,19]. The mechanisms of all these processes and their regulation have been actively studied over the past few years [14,18,20].

Currently, the phenomenon of RNA interference (RNAi) and sRNAs are also considered important regulators of gene expression reprogramming in plant immune responses, pathogen or pest virulence, and communication in plant–microbial interactions [21,22,23,24,25,26,27]. Previous studies showed that miRNAs that induce RNAi are involved in plant response to sap-sucking insects. In melon, 23 conserved miRNA families and 18 cucurbit-specific miRNAs were identified after aphid infestation [28]. Twenty-seven stress-related miRNAs were identified in wheat inoculated with Russian wheat aphids *Diuraphis noxia*; among them conserved miR159 and miR167 [29]. In tobacco plants, 47 miRNAs were differentially expressed, of which 30 were upregulated and 17 downregulated by the phloem-feeding insect whitefly *Bemisia tabaci* exposure [26]. sRNAs are generated by DCLs and function through AGOs to silence target genes [21]. The role of AGO1, AGO4, AGO5, DCL2, and DCL4 has been detected in the regulation of defense responses of Arabidopsis, cotton, and wheat against various aphid species [30,31,32,33]. Although the role of miRNAs in plant defense against insects has been established, the mechanisms of action and mechanisms of regulation of RNAi have not been determined. Nevertheless, it has been established that phytohormones play a regulatory role in genome reprogramming in the processes of RNA interference and DNA (de)methylation during the development of priming [34,35]. Currently, it is recognized that many miRNAs are involved in the regulation of signaling and the synthesis of various phytohormones [36]. However, in the literature, there is scattered information on the participation of phytohormones in the functioning of the RNAi system during the development of plant immunity, especially in the regulation of bacteria-mediated priming [34,36].

PGPBs can be inducers of sRNA expression in plants by triggering ISR, but the mechanism of this process is still poorly understood [34]. Recent works have shown that some bacteria of the genus Bacillus can trigger ISR signaling by regulating the expression of microRNAs through the activation of hormonal signaling pathways [37,38,39,40]. However, these works on the effect of PGPBs on miRNA expression in plants were made during the development of plant resistance against pathogens but not against insects.

This work is focused on studying the role of *B. subtilis* 26D in the development of defensive reactions along the priming pathway in wheat plants against the bird cherry-oat aphid *Rhopalosiphum padi* L. The PGPB successfully colonized the internal tissues of potato, tomato, and wheat plants, stimulated plant growth, and enhanced the defense response of plants against pathogens such as *Phytophthora infestans* and *Stagonospora nodorum*, pests such as Colorado potato beetle *Leptinotarsa decemlineata* and Greenbug aphid *Schizaphis graminum*, and viruses [12,41,42,43,44]. Previously, it was shown that *B. subtilis* 26D produces several metabolites, such as lipopeptides (surfactin) and phytohormones (cytokinins and auxins) [12]. It has been confirmed that surfactin suppresses pathogen growth, possesses aphicidal activity, and activates the ISR against pathogens and pests [12,42].

In this regard, the aim of this work is to study the role of hormonal signaling pathways in the *B. subtilis* 26D strain-mediated triggering of ISR, activation of RNAi system components, and microRNA expression in wheat plants (*Triticum aestivum* L.) colonized by the bird cherry-oat aphid *R. padi*.

Our results suggest that the bacterial strain *B. subtilis* 26D influenced the RNA interference system and miRNA expression in wheat plants through the induction of hormonal signaling pathways during the development of defense responses against the bird cherry-oat aphid.

## 2. Materials and Methods

### 2.1. Research Objects

**Figure 1:**
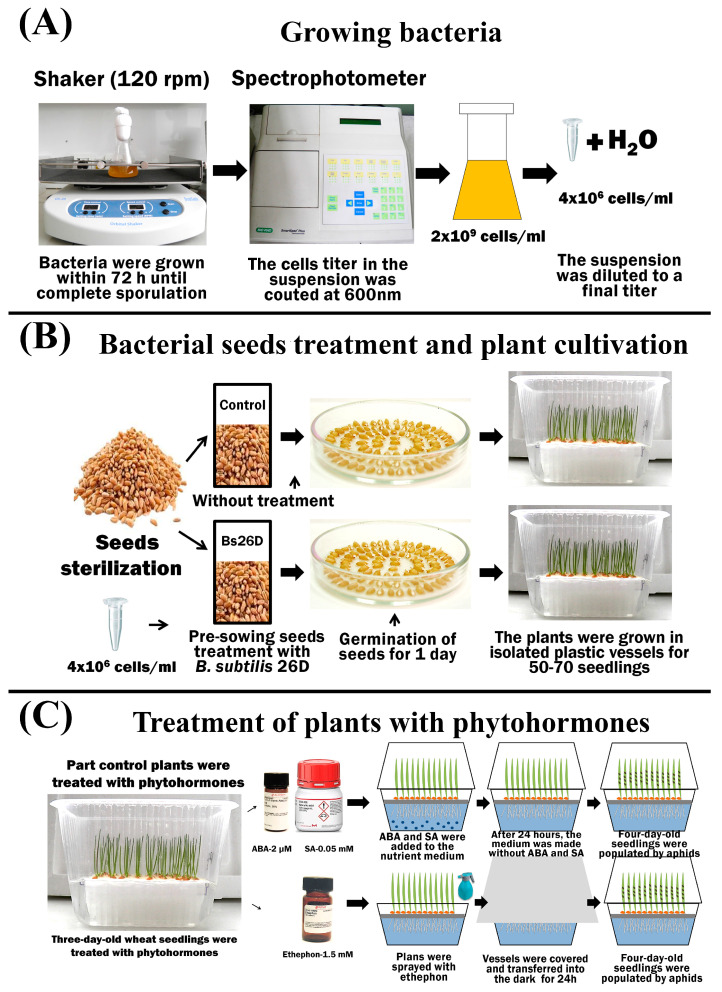
The scheme of the experiment showing the process of growing bacteria in laboratory conditions and measuring the titer of bacterial cells (**A**); sterilization, treatment, and germination of seeds, as well as plant growth conditions (**B**); treatment of plants with phytohormones ABA, SA, and ethephon (**C**).

Bacteria: *B. subtilis* 26D—aerobic, Gram-positive bacteria from the collection of the Laboratory of Biochemistry of Plant Immunity of the Institute of Biochemistry and Genetics Ufa Federal Research Center Russian Academy of Sciences (UFRC RAS) (http://ibg.anrb.ru/wp-content/uploads/2019/04/Katalog-endofit.doc, accessed on 1 November 2023) was used. A liquid lysogenic broth (LB) medium (1% tryptone, 0.5% yeast extract, and 0.5% NaCl) was used to grow bacteria. The bacteria grew at 28 °C for 72 h until complete sporulation on laboratory shakers (Figure 1).

Aphids: The insect population (*Rhopalosiphum padi* L.) was collected in the spring of 2022 from bird cherry plants. The plants grow in the Iglinsky district of the Republic of Bashkortostan (54°50.48′94.0″ N; 56°26.46′09.0″ E) and have never been treated with pesticides. *R. padi* were propagated under controlled laboratory conditions in isolated containers with sterile soil on bread spring wheat (*Triticum aestivum* L.) cultivar Salavat Yulaev under the conditions described previously for *S. graminum* [12].

Plants: In this work, the cultivar Salavat Yulaev was used for experiments, which showed moderate susceptibility to the Greenbug aphid *S. graminum* in earlier work [45]. Seeds were obtained from the Bashkir research Institute of Agriculture—a subdivision of the UFRC RAS.

### 2.2. Experimental Design

The plants were grown hydroponically in insulated plastic containers for each individual treatment option. A 10% Hoagland–Arnon solution was used as a nutrient medium. The plants were grown in a climatic chamber (Binder GmbH, Tuttlingen, Germany) under the conditions described previously for *S. graminum* [12] (Figure 1).

To study molecular and biochemical parameters, plants were grown in large containers with 50–70 seedlings each; to study different types of aphid resistance, plants were grown individually or with 5 seedlings in plastic beakers in a 10% Hoagland–Arnon solution. Each four-day-old plant was colonized by at least 10 aphids. The containers and beakers were insulated with a non-woven porous material to prevent aphid migration. In histograms and tables, “Control” refers to plants that were grown without any treatments and were not infested with aphids. Plants without treatments and colonized by aphids in the tables have the designation “Water”. In each studied variant, 5 aphid-infested and 5 aphid-uninfested plants were taken.

Bacterial treatment: To study the influence of endophytic bacteria on plant defense reactions and growth parameters, experimental wheat seeds were treated before germination with cells of the *B. subtilis* 26D strain in a semi-dry manner in growth-promoting concentrations [12]. The cell titer in the suspension was counted at 600 nm using a SmartSpectm Plus spectrophotometer (BioRad Laboratories, Hercules, CA, USA). The cell titer of the studied cultures was 2 × 10^9^ cells/mL; by adding distilled water, the suspensions were diluted to a final titer of 4 × 10^6^ cells/mL, and the resulting suspensions were used for seed treatment (Figure 1).

Treatment with phytohormones: Phytohormones SA and ABA (Merck KGaA, Sigma-Aldrich, Darmstadt, Germany) were added to the nutrient medium of plants in a final concentration of 0.05 mM and 2 µM, respectively, 24 h before the colonization of aphids. After 24 h, the medium was replaced with Hoagland–Arnon solution without phytohormones (Figure 1). Other plants were sprayed with 1.5 mM solution of 2-chloroethylphosphonic acid (ethephon, ET) (Merck KGaA, Sigma-Aldrich, Darmstadt, Germany) in separate vessels [46] (Figure 1).

### 2.3. Bioassay of the Different Types of Resistance to Aphids—Antibiosis and Endurance

**Figure 2:**
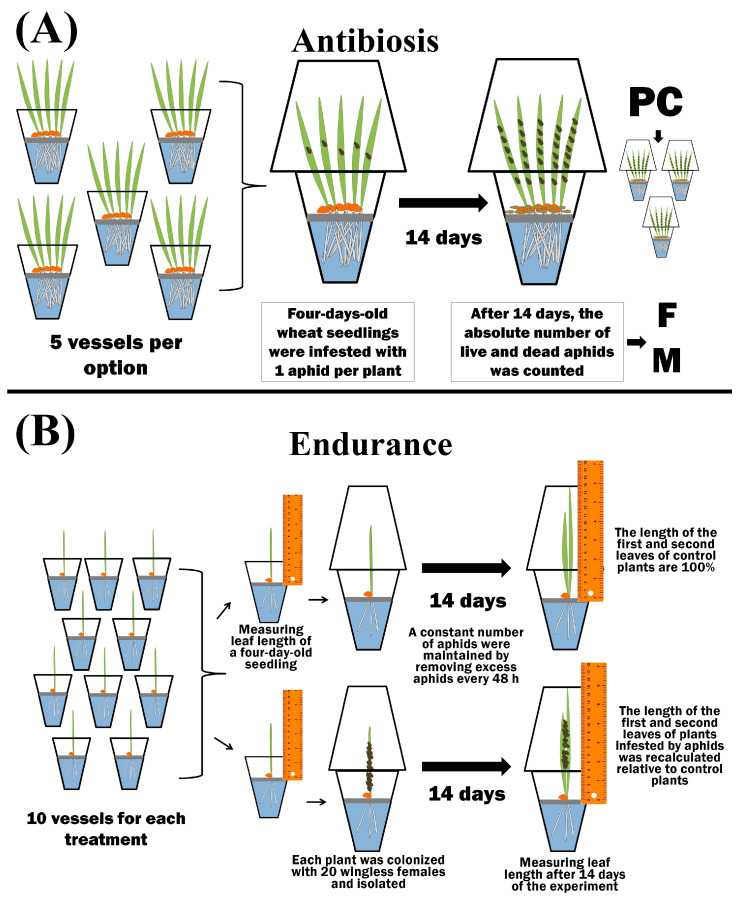
The scheme of bioassay of the different types of resistance to aphids—antibiosis (**A**) and endurance (**B**).

To test the antibiosis, 5 plants were grown in separate isolated plastic beakers for each treatment variant (5 plastic beakers per option) (Figure 2). 

Four-day-old wheat seedlings were infested with 1 aphid per plant. After 14 days, the absolute number of live and dead bird cherry-oat aphids was counted (Figure 2) [47]. The propagation coefficient (PC) was calculated using Equation (1):

(1) $$\mathrm{PC}=\left(\overset{d}{\underset{i=1}{\sum }}F_{i}\right)/d$$

where *F_i_*—denotes the fecundity of one female per *i*-day; *d*—denotes the number of the experiment days (*d* = 14). Fecundity (F) was expressed as the number of nymphs per seedling. For each experimental variant, the average value of PC from 15 repetitions was calculated. Mortality (M) was calculated using Equation (2) and expressed as % of the total number of aphids:

(2) $$M=\left(\overset{n}{\underset{i=1}{\sum }}\frac{m\times 100}{a}\right)/n$$

where *m*—denotes the number of dead aphids, *a* denotes the total number of aphids in one vessel after 14 days of the experiment, and *n*—denotes the number of vessels in one variant of the experiment in which aphids were counted (*n* = 5). Plants were individually grown in isolated plastic beakers to determine endurance (E). Each treatment option contained 10 vessels (Figure 2). The length of four-day-old seedlings was measured from the level of the raft to the tip of the leaf. Then, 20 wingless females were placed on each plant, and the vessel was isolated. During the experiment, excess aphids were removed every 48 h over a two-week period to maintain a constant number of bird cherry-oat aphids (Figure 2). After two weeks, the length of the first and second leaves was measured in both control and aphid-colonized plants. The results of the final measurements were compared with the initial ones [47]. Leaf growth (G_cont_) of control plants was calculated using Equation (3):

(3) $$G_{\mathrm{cont}}=\left(\overset{n}{\underset{i=1}{\sum }}(l_{14i}-l_{i}\right))/n$$

where *l_i_*—denotes the length of the leaf of control 4-day-old plants, *l*_14*i*_—denotes the length of the leaf of the same control plant after 14 days, and *n*—denotes the number of vessels in each variant of the experiment. Leaf growth (G_exp_) of plants treated with bacteria, phytohormones, or colonized by aphids was calculated using Equation (4):

(4) $$G_{\exp}=\left(\overset{n}{\underset{i=1}{\sum }}(l_{e14i}-l_{ei}\right))/n$$

where *l_ei_*—denotes the length of the leaf of 4-day-old plants treated with bacteria, phytohormones, or colonized by aphids, *l_e14i_*—denotes the length of the leaf of the same experimental plant after 14 days, and *n*—denotes the number of vessels in each variant of the experiment. The average endurance (E) in each experimental variant for both the 1st and 2nd leaves was calculated using Equation (5):

E = (G_exp_ × 100)/G_cont_ (5)

Endurance was expressed in % of leaf growth compared to unpopulated control (Figure 2).

### 2.4. Bioassay of the Biochemical Parameters

The content of hydrogen peroxide (H_2_O_2_) and the activity of enzymes—peroxidase (POD) and catalase (CAT)—were carried out according to standard methods. For this, plant material was fixed in liquid nitrogen 24, 72, and 144 h after plant colonization by bird cherry-oat aphids. Plant material was extracted in 0.05 M solution of Na-phosphate buffer (PB), pH 6.2 (1:5 weight/volume) at 4 °C for 30 min. Then, the plant extract was centrifuged at 15,000× *g* for 15 min (5415 K Eppendorf, Hamburg, Germany), and the supernatant was used for further analyses. The concentration of H_2_O_2_ in the supernatant was determined using xylenol orange in the presence of Fe^2+^, where hydroperoxides are reduced by ferrous ions in acid solution forming a ferric product–xylenol orange complex, detected spectrophotometrically at 560 nm [48]. The POD activity was detected by a microassay in 96-well plates (Corning-Costar, Glendale, AZ, USA) by the oxidation of (*o-*) phenylenediamine in the presence of 25 μL of 0.0016% H_2_O_2_ solution. Optical density at 490 nm was measured on a Benchmark Microplate Reader spectrophotometer (Bio-Rad Laboratories, Hercules, CA, USA) [47]. The enzyme activity was expressed in optical density/mg of protein per minute, which corresponded to the amount of oxidized substrate causing an increase in optical density in 1 min. CAT activity was determined by a microassay in 96-well plates by the assay based on the ability of H_2_O_2_ to form a stable-colored complex with molybdate salts [47]. In a well, 150 μL 0.03% H_2_O_2_ (or water in the control) was mixed with 20 μL supernatant. The reaction was stopped by the addition of 75 μL 4% ammonium molybdate after 1 min. Optical density was measured at 405 nm on a Benchmark Microplate Reader spectrophotometer. CAT activity was calculated using a calibration curve and expressed in μM H_2_O_2_/mg of protein per min. Protein content was determined with the Bradford method.

### 2.5. Gene Expression Analysis

Leaves from five plants per biological replication were collected and fixed in liquid nitrogen 6, 24, 72, and 144 h after population by aphids. Total wheat RNA was extracted using Lira^®^ (Biolabmix, Moscow, Russia) according to the manufacturer’s instructions. For cDNA synthesis, the method described in an earlier work was used [44]. Primers for real-time polymerase chain reaction (real-time PCR) were devised using the web tool PrimerQuest™ Tool (http://eu.idtdna.com/Scitools/Applications/Primerquest, accessed on 1 November 2023) (Integrated DNA Technologies, Inc., Coralville, IA, USA). The sequences of all the primers are presented in Table S1 (Supplementary Materials) for genes encoding enzymes of phytohormones biosynthesis, the TF of these hormonal signaling pathways and PR-proteins, in Table S2 (Supplementary Materials) for genes encoding Dicer-like proteins *DCL2* and *DCL4*, and four genes encoding the Argonaute proteins *AGO1*, *AGO2*, *AGO4,* and *AGO5*. The annealing temperature of the primers was 60 °C. A melting curve analysis was conducted to determine the specificity of the reaction (at 95 °C for 15 s, 60 °C for 1 min, and 95 °C for 15 s). The efficiency of the primers was determined using a series of cDNA dilutions (10-fold). To standardize the data, wheat gene *TaRLI* (RNaseLinhibitor-like) (Tables S1 and S2 Supplementary Materials) was used as a positive internal control for the real-time PCR analysis for both cases. Real-time PCR was performed on a “DNA amplifier in real time” CFX96 Touch with fluorescent detection (BioRad Laboratories, Hercules, CA, USA). For detection, a set of reagents, EvaGreen I (Synthol, Moscow, Russia), was used. In order to quantify the relative gene expression, the delta–delta Ct method was performed as described earlier [44]. Three independent biological and three technical replications were performed for each experiment.

### 2.6. Isolation of Plants miRNA and miRNA Expression Analysis

Plant material for microRNA isolation was fixed 6, 24, 72, and 144 h after aphid colonization of plants. MicroRNA was extracted from 100 mg of control and aphid-infested wheat seedlings using a “Total RNA and small RNA isolation kit” (Biolabmix, Moscow, Russia), containing reagents for phenol-chloroform extraction of nucleic acids and their selective sorption on a silicon membrane. RNA concentration was determined at 260 nm using an ND-1000 spectrophotometer (NanoDrop Technologies LLC, Wilmington, DE, USA). Polyadenylation of isolated from plants and purified microRNA was carried out in a reaction volume containing 0.3 µg RNA and 5 U *Escherichia coli* poly(A) polymerase (New England Biolabs, Ipswich, MA, USA) at 37 °C for 1 h in a microtube thermostat [49]. First-strand miRNA cDNA was generated by mixing 500 ng of poly(A) tailed RNA and 0.5 µg of RTQ primer, followed by the addition of reverse transcriptase (M-MLV, Synthol, Moscow, Russia). Then, the mixture was incubated at 37 °C for 1 h.

miRNAs were performed using microRNA-specific forward primers and the universal reverse primer with EvaGreen I intercalating dye (Synthol, Moscow, Russia). Real-time PCR was performed using a “DNA amplifier in real time” CFX96 Touch with fluorescent detection (Bio-Rad Laboratories, Hercules, CA, USA). The constitutively expressed 5S rRNA gene was used as internal control for normalize the results of the miRNA expressions. Primers for real-time PCR were devised using a web database https://www.pmiren.com (accessed on 1 November 2023). The primer sequences are all presented in Table S3 (Supplementary Materials). The presence of only a single peak on the thermal dissociation (Tm) curve confirmed the specificity of primers, which was generated by thermal denaturing. The relative gene expression was calculated using the delta–delta Ct method [44]. Three independent biological and three technical replicates were performed for each experiment.

### 2.7. Statistical Analysis

All experiments were repeated three times with a different number of biological repetitions. Thus, for molecular and biochemical parameters, 3 replicates were used; for the antibiosis test, 5 replicates were employed; and for calculating endurance, 10 biological replicates were applied in one experiment. Experimental data were expressed as mean ± SE, calculated using MS Excel in all treatments. The significance of the differences was assessed by ANOVA followed by Duncan’s test (*p* ≤ 0.05) using STATISTICA 10.0 software (version STA999K347150-W, Tulsa, OK, USA). The treatment variants and the number of repetitions are indicated in the tables and figures.

## 3. Results

### 3.1. The Plant-Mediated Effect of the B. subtilis 26D Strain on the Different Types of Defense against Aphids—Antibiosis and Endurance

Bacteria can have a plant-mediated effect on the viability of the pest, increasing various types of plant resistance against aphids—antibiosis and endurance. In the present experiments, bird cherry-oat aphid *R. padi* inhibited the growth of the 1st and 2nd leaves to 77 and 80%, respectively, compared with the control plants non-infested with aphids (100%) of the moderately susceptible cultivar Salavat Yulaev (Table 1). Pre-sowing treatment of wheat seeds with the *B. subtilis* 26D strain accelerated the growth of the 1st and 2nd leaves of wheat during aphid colonization; such plants grew even better than control plants by 2–6% (Table 1). Thus, the *B. subtilis* 26D strain, influencing plant growth, formed a certain plant tolerance to the pest. 

In addition, the *B. subtilis* 26D strain indirectly increased the mortality of *R. padi* by 32% and reduced their fecundity and reproduction rate (propagation coefficient) when aphids fed on wheat plants treated with bacteria by approximately 1.5 and 2.2 times, respectively (Table 1).

### 3.2. The Plant-Mediated Effect of the B. subtilis 26D Strain on Changes in the Redox Status of R. padi-Infested Wheat Plants

#### 3.2.1. The Content of Hydrogen Peroxide and Activity of Redox Enzymes in Wheat Plants

The plant-mediated effect of endophytes of *Bacillus* spp. on plant endurance and aphid vitality indicators may be connected with the triggering of induced systemic resistance (ISR) in plants [8,47]. Bacteria, during the development of ISR, can affect the accumulation of ROS, both locally and systemically [8].

**Figure 3:**
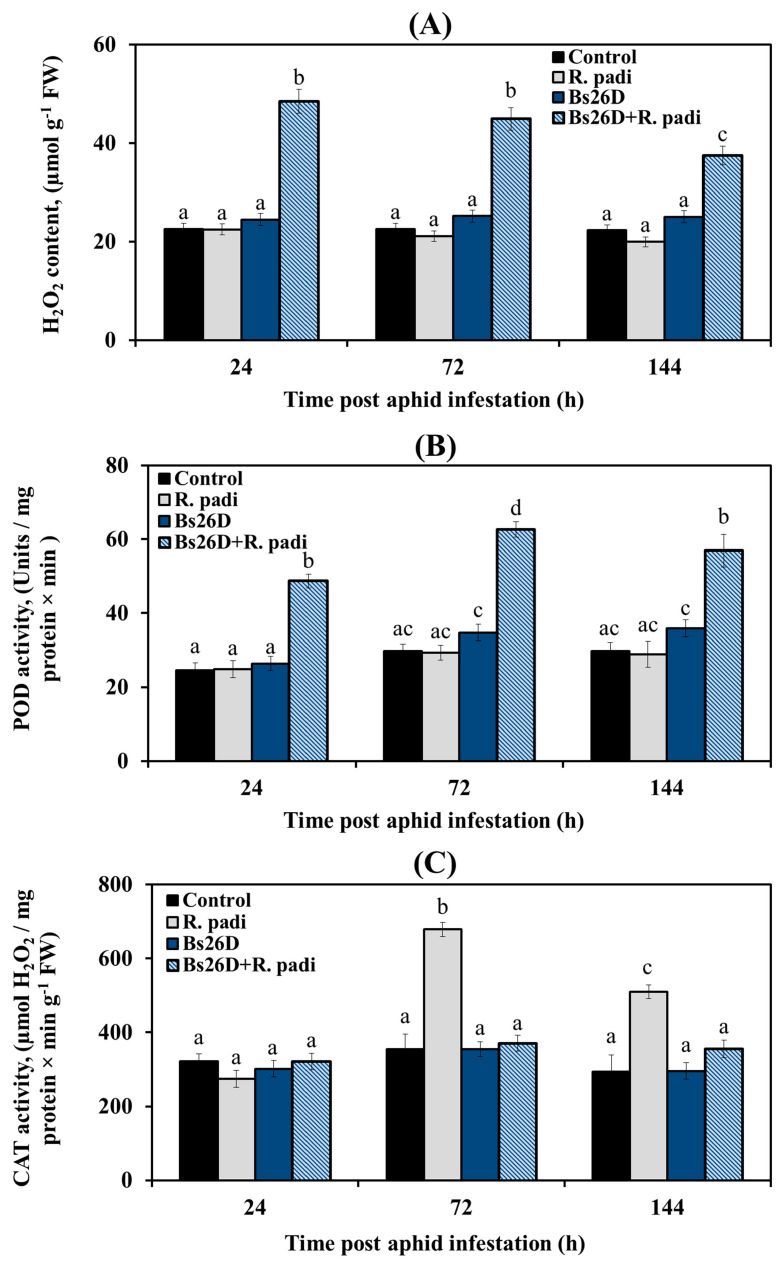
Influence of *B. subtilis* 26D strain (Bs26D) on the hydrogen peroxide (H_2_O_2_) content (**A**), peroxidase activity (POD) (**B**), and catalase activity (CAT) (**C**) of wheat plants infested with bird cherry-oat aphid *R. padi*. The samples are indicated as follows: Control—non-bacterized plants and unpopulated with aphids; Bs26D—plants treated with the *B. subtilis* 26D strain before sowing; *R. padi*—plants populated with aphids; Bs26D + *R. padi*—bacterized plants and populated with aphids. Figures present means ± SE (*n* = 6). Columns of each histogram marked with different letters represent the mean values that are statistically different from each other according to Duncan’s test (*p* ≤ 0.05).

These results showed that H_2_O_2_ content did not change; peroxidase (POD) activity did not change 24, 72, and 144 h after bird cherry-oat aphid infestation of control wheat plants (Figure 3A,B). The catalase (CAT) activity significantly increased by approximately two times 24 and 72 h after bird cherry-oat aphid colonization of control wheat plants (Figure 3C). In wheat plants treated with the *B. subtilis* 26D strain and infested with *R. padi*, the H_2_O_2_ content and POD activity increased sharply, while CAT activity did not change compared to the control ones (Figure 3), which may have determined the resistance of such plants against the pest.

#### 3.2.2. Expression of Genes Relating to Plant Hormone Signaling Pathways

To determine the ability of the *B. subtilis* 26D strain to trigger and regulate the ISR of wheat against *R. padi*, the plants’ gene expression encoding enzymes of phytohormones biosynthesis SA, JA, ethylene, and ABA was studied. The gene expression encoding transcription factors (TF) of these hormonal signaling pathways and PR-proteins, markers of SA, JA ethylene, and ABA signaling pathways were also studied.

We studied the expression of genes involved in the SA biosynthesis (isochorismate synthase, *TaICS*), in the JA biosynthesis (lipoxygenase, *TaLOX*), in the biosynthesis of ethylene (aminocyclopropane synthase, *TaACS*) and *TaNCED* (9-cis-epoxycarotenoid dioxygenase) gene, which controls the rate-limiting step of ABA biosynthesis [13,14,50,51,52].

**Figure 4:**
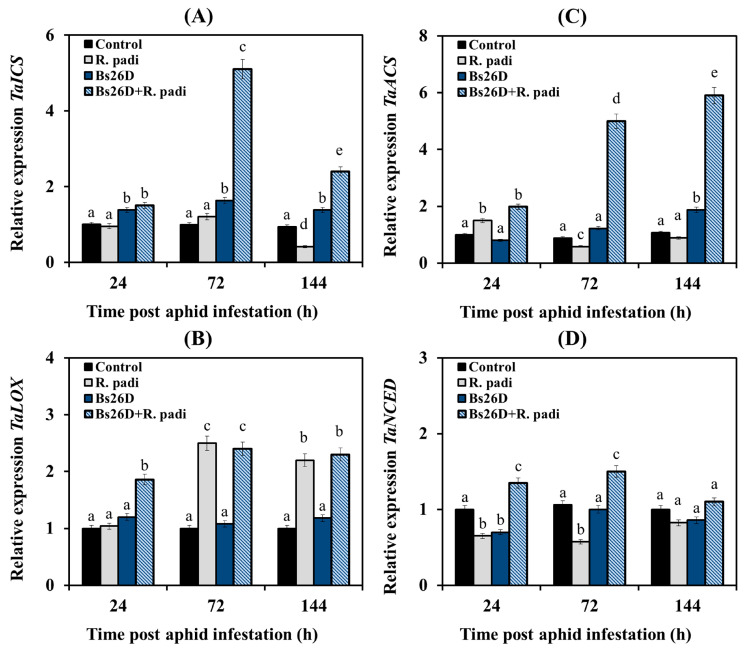
Influence of *B. subtilis* 26D strain (Bs26D) on the relative expression of genes involved in the SA biosynthesis, *TaICS* (**A**), the JA biosynthesis, *TaLOX* (**B**), the ethylene biosynthesis, *TaACS* (**C**) and the ABA biosynthesis, *TaNCED* (**D**) in wheat plants infested with bird cherry-oat aphid *R. padi*. The samples are indicated as follows: Control—non-bacterized plants and unpopulated with aphids; Bs26D—plants treated with the *B. subtilis* 26D strain before sowing; *R. padi*—plants populated with aphids; Bs26D + *R. padi*—bacterized plants and populated with aphids. Figures present means ± SE (*n* = 6). Columns of each histogram marked with different letters represent the mean values that are statistically different from each other according to Duncan’s test (*p* ≤ 0.05).

Experimental results showed that the transcription of the *TaICS* and *TaNSED* genes did not change or decrease in non-bacterized wheat plants of the moderately susceptible cultivar Salavat Yulaev after aphid colonization compared to the control (Figure 4A,D). The mRNA abundance of the *TaACS* gene increased 24 h after bird cherry-oat aphid infestation of control wheat plants, and then the transcript levels of this gene decreased (Figure 4C). The mRNA abundance of the *TaLOX* gene increased in non-bacterized plants 72 and 144 h after colonization by aphids (Figure 4B).

The pre-sowing treatment of wheat seeds with the *B. subtilis* 26D strain led to a significant accumulation of *TaICS* gene transcripts in wheat plants after aphid colonization compared to the control (Figure 4A). Thus, 72 h after aphid infestation, the mRNA content of the *TaICS* gene increased by 5 times compared with the control in these plants (Figure 4A). The treatment of wheat seeds with *B. subtilis* 26D resulted in an increase in the transcript levels of the *TaNSED* gene by 2 times compared with the non-bacterized plants colonized with aphids (Figure 4D). The treatment of wheat seeds with *B. subtilis* 26D led to significant accumulation of the transcript levels of the *TaACS* gene by 2–6 times in wheat plants 24, 72, and 144 h after aphid colonization compared to the control (Figure 4C). The mRNA abundance of the *TaLOX* gene increased in bacterized plants 24, 72, and 144 h after infestation by *R. padi* (Figure 4B).

In this work, we studied the expression of the TF genes. The gene of the SA signaling pathway *TaWRKY13* is an ortholog of the Arabidopsis gene *AtWRKY70* [53]; TF of the ethylene signaling pathway *TaEIN3* (Ethylene-Insensitive3) is an ortholog of the Arabidopsis gene *AtEIN3* and TF of the primary response to ethylene *TaERF1* (Ethylene Response Factor1) activates the ERF branch of the JA signaling pathway and is responsible for the integration of ethylene signaling pathways and JA [3]; TF of the ABA signaling pathway *TaABI5* (Abscisic Acid Insensitive5) is positive regulator in early post-invasive resistance [51].

**Figure 5:**
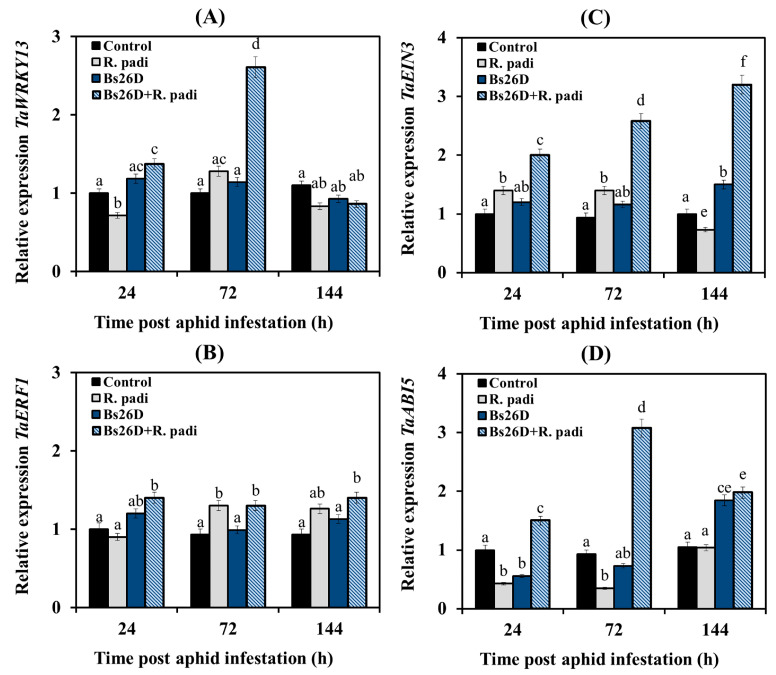
Influence of the *B. subtilis* 26D strain (Bs26D) on the relative expression of genes the SA signaling pathway, *TaWRKY13* (**A**), the JA signaling pathway, *TaERF1* (**B**), the ethylene signaling pathway, *TaEIN3* (**C**) and the ABA signaling pathway, *TaABI5* (**D**) in wheat plants infested with bird cherry-oat aphid *R. padi*. The samples are indicated as follows: Control—non-bacterized plants and unpopulated with aphids; Bs26D—plants treated with the *B. subtilis* 26D strain before sowing; *R. padi*—plants populated with aphids; Bs26D + *R. padi*—bacterized plants and populated with aphids. Figures present means ± SE (*n* = 6). Columns of each histogram marked with different letters represent the mean values that are statistically different from each other according to Duncan’s test (*p* ≤ 0.05).

Our results showed that the transcription of the *TaWRKY13* gene decreased in non-bacterized wheat plants at 24 and 144 h after *R. padi* colonization compared to the control, and transcript levels of this gene increased insignificantly at 72 h feeding of aphids on plants (Figure 5A). The transcript level of the *TaERF1* gene did not change in such plants at 24 h after aphid infestation and then slightly increased at 72 and 144 feeding of aphids on plants compared to the control (Figure 5B). The transcript level of the *TaEIN3* gene in non-bacterized wheat plants slightly increased at 24 and 72 h after aphid population and then decreased at 144 after aphid colonization compared to the control (Figure 5C). The transcript level of the *TaABI5* gene decreased in non-bacterized wheat plants at 24 and 72 h after aphid infestation compared to the control, and then the transcript level of this gene was restored to control values (Figure 5D).

The pre-sowing treatment of wheat seeds with the *B. subtilis* 26D strain slightly induced the mRNA abundance of the TF gene of the JA signaling pathway *TaERF1* and significantly increased by approximately three times in the transcript level of the TF genes of the SA signaling pathway *TaWRKY13*, the ethylene signaling pathway *TaEIN3,* and the ABA signaling pathway *TaABI5* (Figure 5).

**Figure 6:**
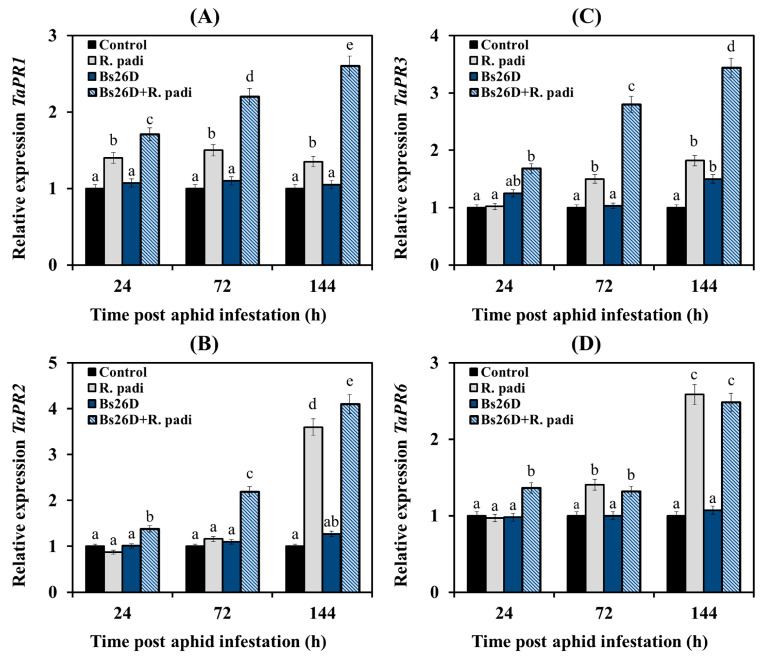
Influence of the *B. subtilis* 26D strain (Bs26D) on the relative expression of SA-dependent genes *TaPR1* (**A**) and *TaPR2* (**B**), ethylene-dependent gene *TaPR3* (**C**), and JA-dependent gene *TaPR6* (**D**) in wheat plants infested with bird cherry-oat aphid *R. padi*. The samples are indicated as follows: Control—non-bacterized plants and unpopulated with aphids; Bs26D—plants treated with the *B. subtilis* 26D strain before sowing; *R. padi*—plants populated with aphids; Bs26D + *R. padi*—bacterized plants and populated with aphids. Figures present means ± SE (*n* = 6). Columns of each histogram marked with different letters represent the mean values that are statistically different from each other according to Duncan’s test (*p* ≤ 0.05).

In our work, the expression of the *TaPR1* and *TaPR2* genes, which are markers of the SA signaling pathway, the *TaPR3* gene, which is the marker of the ethylene signaling pathway, and the *TaPR6* gene, which is the marker of the JA signaling pathway, were studied [8,13]. Experimental results showed that the mRNA abundance of the *TaPR1* gene slightly increased in non-bacterized and infested with bird cherry-oat aphid wheat plants (Figure 6A). The transcript level of the *TaPR2* gene did not change 24 and 72 h after aphid colonization and then significantly increased by 3.6 times 144 h after the infestation of control plants with *R. padi* (Figure 6B).

The transcript level of the *TaPR3* and *TaPR6* genes did not change at 24 h feeding of aphids on plants, slightly increased 72 h after aphid colonization, and significantly increased by 1.8 and 2.6 times, respectively, 144 h after the infestation of control plants with *R. padi* (Figure 6C,D).

The pre-sowing treatment of wheat seeds with the *B. subtilis* 26D strain led to a significant accumulation of mRNA of *TaPR1*, *TaPR2*, *TaPR3,* and *TaPR6* genes in wheat plants colonized with bird cherry-oat aphids compared to the control (Figure 6). The effect of plant treatments with bacterial strain *B. subtilis* 26D on the expression of *TaPR1* and *TaPR3* genes was different from the effect on the expression of the *TaPR2* and *TaPR6* genes (Figure 6). The *B. subtilis* 26D strain significantly affected the expression of *TaPR1* and *TaPR3* genes throughout the experiment (Figure 6A,C). A significant effect of treatment with the *B. subtilis* 26D strain on the transcript level of the *TaPR2* and *TaPR6* genes was detected only at the initial stage feeding of aphids on plants, 24 and 72 h after colonization for the *TaPR2* gene and 24 h after infestation for the *TaPR6* gene (Figure 6B,D). There was no effect of bacterial treatment on the expression of these genes after 144 h of feeding (Figure 6B,D).

Thus, the colonization of wheat plants with bird cherry-oat aphids led to a slight induction of JA/ethylene signaling pathways since the transcription of genes of these hormonal signaling pathways—*TaLOX*, *TaERF1*, *TaPR6,* and *TaACS*, *TaEIN3*, *TaPR3* was activated (Figure 4, Figure 5 and Figure 6). The treatment of wheat seeds with the *B. subtilis* 26D strain led to a strong induction of SA and ethylene signaling pathways since a significant increase in the transcript level of the *TaICS TaWRKY13*, *TaPR1*, *TaPR2,* and *TaACS*, *TaEIN3*, *TaPR3* genes was found in bacterized plants colonized with aphids (Figure 4, Figure 5 and Figure 6). 

In addition, treatment with the *B. subtilis* 26D strain led to the induction of JA and ABA signaling pathways; the accumulation of transcripts of the *TaLOX*, *TaERF1*, *TaPR6,* and *TaNCED*, *TaABI5* genes was detected in bacterized plants colonized with aphids (Figure 4, Figure 5 and Figure 6).

### 3.3. The Plant-Mediated Effect of B. subtilis 26D on the Expression of the RNA Interference System Genes AGO and DCL in R. padi-Infested Wheat Plants

In this work, we studied the expression of two genes encoding Dicer-like proteins, *DCL2* and *DCL4*, which are involved in the regulation of plant immunity against viruses and insects [21,54]. We also studied the expression of four genes encoding the Argonaute proteins *AGO1*, *AGO2*, *AGO4,* and *AGO5*, which play a role in the interaction of plants and microorganisms, in the development of protective reactions in response to stress and are involved in RdDM [21]. 

**Figure 7:**
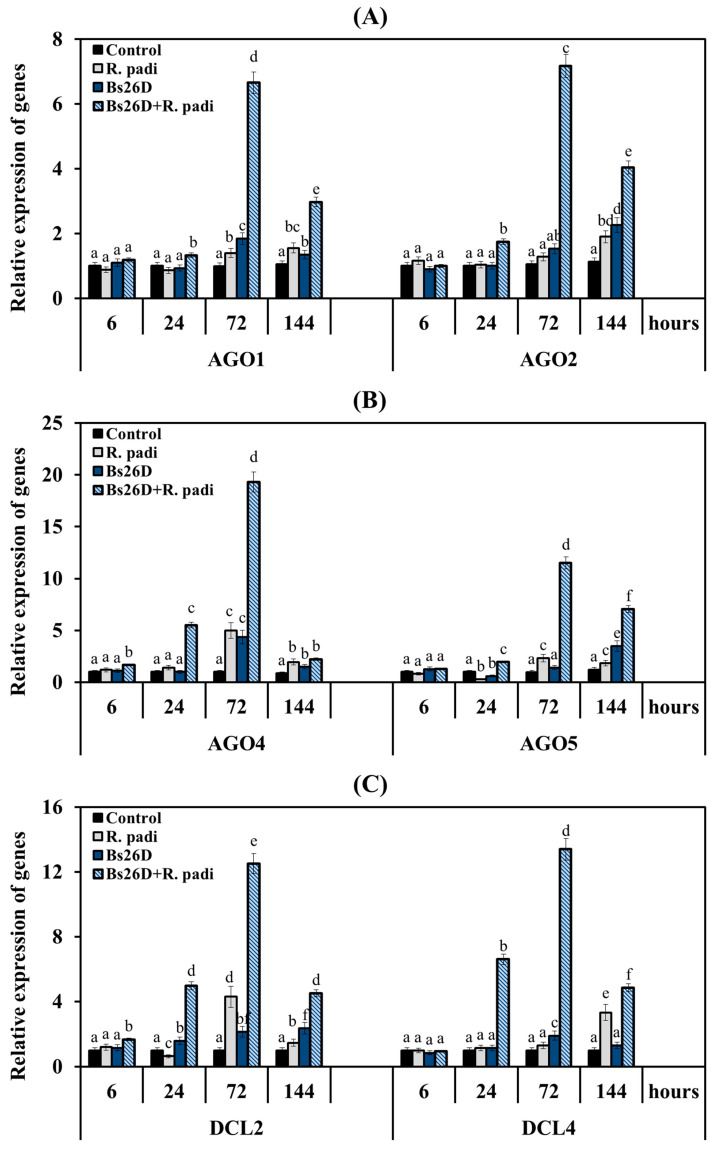
Influence of the *B. subtilis* 26D strain (Bs26D) on the relative expression of genes encoding RNAi enzymes *AGO1* and *AGO2* (**A**), *AGO4* and *AGO5* (**B**), *DCL2* and *DCL4* (**C**) in wheat plants infested with bird cherry-oat aphid *R. padi*. The samples are indicated as follows: Control—non-bacterized plants and unpopulated with aphids; Bs26D—plants treated with the *B. subtilis* 26D strain before sowing; *R. padi*—plants populated with aphids; Bs26D + *R. padi*—bacterized plants and populated with aphids. Figures present means ± SE (*n* = 6). Columns of each histogram marked with different letters represent the mean values that are statistically different from each other according to Duncan’s test (*p* ≤ 0.05).

Analysis of gene expression of RNAi system enzymes in non-bacterized wheat plants of the moderately susceptible cultivar Salavat Yulaev showed that the transcripts level of all six genes did not change or decreased at the initial stage feeding of bird cherry-oat aphids 6 and 24 h after plant colonization, and then the 1.3- to 5-fold increase in mRNA content of these genes was observed 72 and 144 h after the infestation of control plants with *R. padi* (Figure 7). 

The most significant transcript accumulation was found in the *AGO4*, *AGO5,* and *DCL2* genes by 5, 2.3, and 4.3 times, respectively, compared to the control 72 h after the colonization of non-bacterized plants with aphids (Figure 7). The maximum accumulation of the transcript level of the *DCL4* gene by 3.2 times compared to the control in non-bacterized plants inhabited by *R. padi* was detected after 144 h feeding of aphid (Figure 7).

The pre-sowing treatment of wheat seeds with the *B. subtilis* 26D strain led to an earlier and larger accumulation of mRNA for all six genes in aphid-infested plants (Figure 7). The treatment of the *B. subtilis* 26D strain slightly increased the transcript level of the *AGO1* gene 6 and 24 h feeding of aphid and then significantly increased by 6.7 and 3 times at 72 and 144 h after the colonization, respectively, compared to the control (Figure 7A). The treatment of the *B. subtilis* 26D strain significantly increased the mRNA abundance of the *AGO2* gene by 1.8, 7.2, and 4 times compared to the control at 24, 72, and 144 h after the infestation of control plants with *R. padi*, respectively (Figure 7A). The treatment of the *B. subtilis* 26D strain significantly increased the mRNA content of the *AGO4* gene throughout the experiment from 1.7- to 19-fold compared to the control in aphid-infested plants (Figure 7B). The greatest effect of the treatment of the B. subtilis 26D strain on the expression of the *AGO4* gene was found after 24 and 72 h of aphid colonization of plants; transcripts of this gene were increased by 5.5 and 19 times, respectively, compared to the control (Figure 7B). The treatment of the *B. subtilis* 26D strain significantly increased the mRNA content of the *AGO5* gene throughout the experiment from 1.3- to 11.5-fold compared to the control in aphid-infested plants (Figure 7B). The greatest effect of the treatment of the *B. subtilis* 26D strain on the expression of the *AGO5* gene was found after 72 and 144 h of aphid infestation of plants; transcripts of this gene were increased by 11.5 and 7 times, respectively, compared to the control (Figure 7B). The treatment of the *B. subtilis* 26D strain significantly increased the transcript level of *DCL2* and *DCL4* genes by 5 times or more 24 h after aphid colonization of plants (Figure 7C). The greatest effect of the treatment of the *B. subtilis* 26D strain on the expression of the *DCL2* and *DCL4* genes was found after 72 h feeding of aphids on plants; transcripts of these genes were increased by 12.5 and 13.4 times, respectively, compared to the control (Figure 7C). It is worth noting that the effect of the treatment of the *B. subtilis* 26D strain on the expression of the *AGO4* and *DCL2* was detected after just 6 h feeding of aphids on plants; transcripts of these genes were increased by approximately 2 times compared to the control (Figure 7B,C).

Thus, the *B. subtilis* 26D strain influenced the expression of all the studied genes of the RNA interference machine, but the bacterium had the strongest effect on the expression of the *AGO4*, *AGO5* and *DCL2*, *DCL4* genes. 

### 3.4. The Effect of Phytohormones on the Expression of the RNA Interference System Genes AGO and DCL in R. padi-Infested Wheat Plants 

To determine the action mechanism of *B. subtilis* 26D on the gene expression of RNAi system enzymes, we treated the plants with phytohormones SA, ABA, and the chemical precursor of ethylene, ethephon (ET), since it was these hormonal signaling pathways that the *B. subtilis* 26D strain induced in aphid-infested plants (Figure 4, Figure 5 and Figure 6). First, we determined the effect of phytohormonal treatment on two types of plant resistance to aphids—antibiosis and endurance (Table 2).

The results of this work showed that the treatment of plants with SA and ET had a similar effect on aphid viability indicators (Table 2). Plant treatment with SA and ET resulted in an increase in the mortality of *R. padi* by 36 and 38%, a reduction in their fecundity by 39 and 42%, respectively, and a decrease in their propagation coefficient by approximately 2 times compared to untreated aphid-infested plants (Table 2). The treatment of plants with ABA had a stronger effect on aphid viability indicators than SA and ET (Table 2). Plant treatment with ABA resulted in an increase in the mortality of *R. padi* by 45%, a reduction in their fecundity by 58%, and a decrease in their propagation coefficient by approximately 3 times compared to untreated aphid-infested plants (Table 2).

Furthermore, plant treatment with SA and ET restored the growth of the 1st and 2nd leaves of wheat during aphid feeding in a similar manner by 21–22 and 13–16%, respectively, compared to untreated aphid-infested plants (Table 2). The influence of ABA on this indicator was less significant. Treatment of plants with ABA restored the growth of the 1st leaf by 8% and the 2nd leaf by 2% compared to untreated aphid-infested plants (Table 2). Thus, the phytohormones SA, ABA, and ethylene increased the resistance of wheat plants to bird cherry-oat aphid *R. padi*.

Analysis of gene expression of RNAi system enzymes in phytohormones-treated plants showed that the treatments with SA, ET, and ABA had a weak effect on the expression of the *AGO1* and *AGO2* genes during feeding of aphids on plants with some exceptions (Table 3). The treatment of plants with SA and ET led to the accumulation of *AGO1* gene transcripts by 1.7 and 1.5 times, respectively, compared to the control only 24 h after *R. padi* colonization of plants (Table 3). Treatment of plants with ABA increased the mRNA content of the *AGO1* gene by 4 times compared to the control only 144 h after colonization of plants by *R. padi* (Table 3). Treatment of plants with SA did not affect the transcription of the *AGO2* gene (Table 3). Treatment of plants with ET increased the mRNA content of the *AGO2* gene only 24 and 72 h after colonization of plants by aphids, and treatment of plants with ABA increased the mRNA abundance of the *AGO2* gene only 72 and 144 h after population of plants by *R. padi* compared to untreated aphid-infested plants (Table 3).

The treatments with SA, ET, and ABA had a strong effect on the expression of the *AGO4* and *AGO5* genes during the feeding of aphids on plants, with some exceptions (Table 3). Treatment with ET increased the expression of the *AGO4* gene throughout the experiment by 2–11 times compared to the control (Table 3). Treatment of plants with SA increased the mRNA content of the AGO4 gene only 24 and 144 h after colonization of plants by aphids, and treatment of plants with ABA increased the mRNA abundance of the *AGO4* gene only 144 h after population of plants by *R. padi* compared to untreated aphid-infested plants (Table 3).

Increased expression of the *AGO5* gene compared to untreated aphid-infested plants at the initial stage of *R. padi* feeding after 24 h of colonization was found in plants treated with SA, ET, and ABA (Table 3). At the late stage of aphid feeding, after 144 h of colonization, an increase in *AGO5* gene expression was found in plants treated with only ET and ABA (Table 3). Treatment with ET increased the mRNA content of the *AGO5* gene by 13.2 times; ABA treatment increased the mRNA abundance of the *AGO5* gene by 9.7 times compared to the control after 144 h of colonization (Table 3). 

All three hormones increased the expression of the *DCL2* gene by 4–6 times compared to the control in plants infested with aphids (Table 4). However, treatment with SA increased the mRNA content of the *DCL2* gene only after 72 and 144 h, ABA treatment increased the mRNA abundance of this gene after 72 and 144 h, and treatment with ET increased the expression of the DCL2 gene only after 24 and 144 h of aphid feeding (Table 4).

The treatment with ET increased *DCL4* gene expression in plants after 6, 24, and 72 h of *R. padi* feeding (Table 4). SA and ABA affected the expression of this gene at a later time. Thus, treatment with SA increased the transcript level of the *DCL4* gene by 2 and 5 times 72 and 144 h after plant colonization by aphids, respectively (Table 4). Treatment of plants with ABA increased the mRNA content of the *DCL4* gene by 6.6 times 144 h after aphid colonization of plants (Table 4).

Thus, our results showed that the phytohormones SA, ethylene, and ABA regulate the gene expression of RNAi system enzymes in wheat plants during the development of defensive reactions against bird cherry-oat aphid *R. padi*.

### 3.5. B. subtilis 26D, SA, ABA, and Ethylene Regulate miRNA Expression in a Similar Manner in R. padi-Infested Wheat Plants

**Figure 8:**
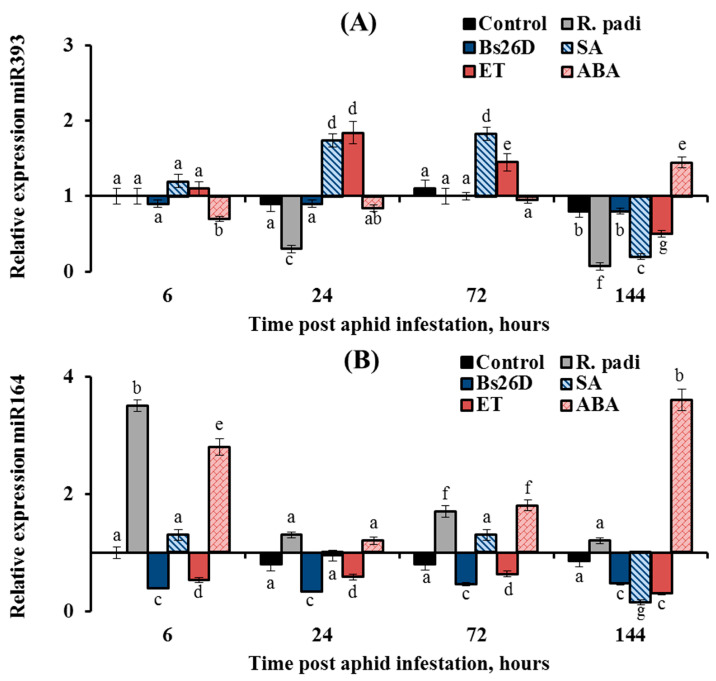
Influence of the *B. subtilis* 26D strain (Bs26D) and phytohormones salicylic acid (SA), abscisic acid (ABA), and ethephone (ET) on the relative expression miRNAs miR393 (**A**), miR164 (**B**) in wheat plants infested with bird cherry-oat aphid *R. padi*. The samples are indicated as follows: Control—non-bacterized plants and unpopulated with aphids; Bs26D—plants treated with the *B. subtilis* 26D strain before sowing; *R. padi*—non-bacterized and untreated plants populated with aphids; SA—plants treated with salicylic acid; ET—plants treated with ethephone; ABA—plants treated with abscisic acid. Figures present means ± SE (*n* = 6). Columns of each histogram marked with different letters represent the mean values that are statistically different from each other according to Duncan’s test (*p* ≤ 0.05).

**Figure 9:**
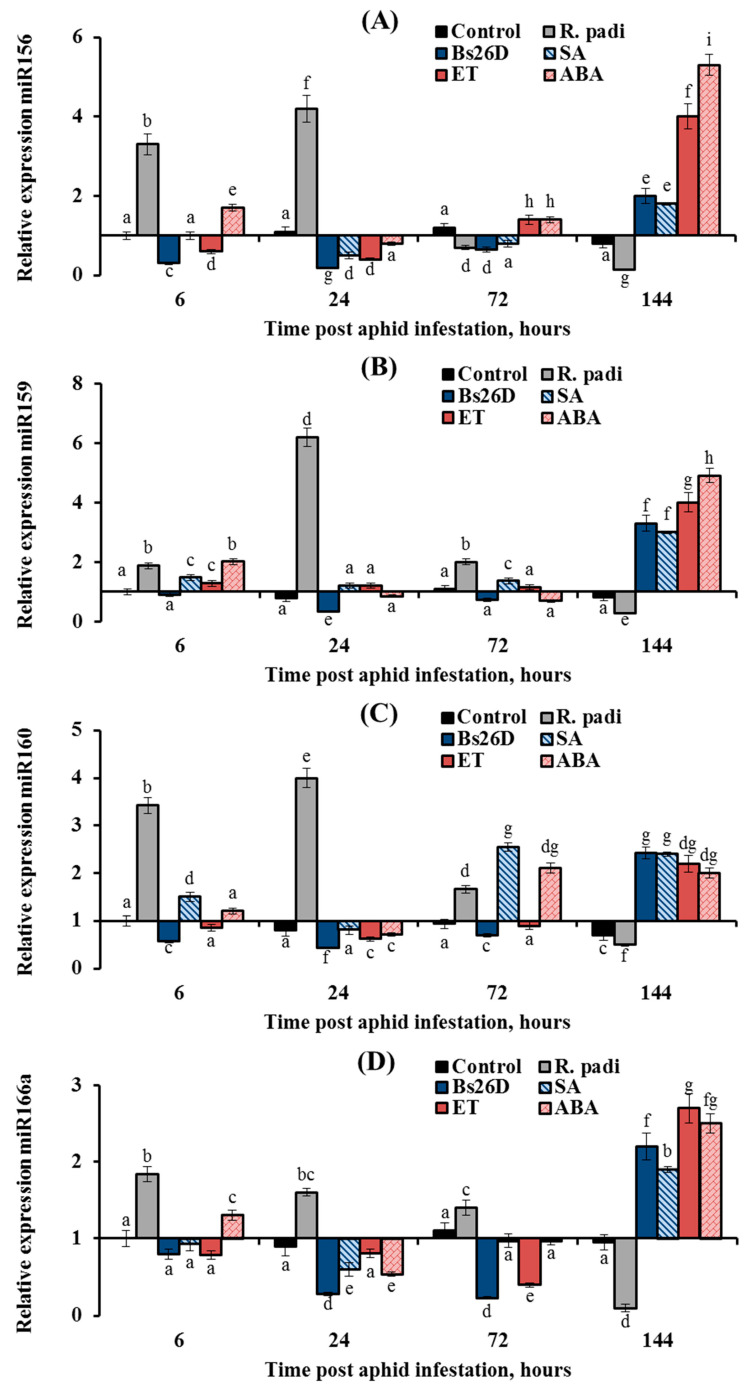
Influence of the *B. subtilis* 26D strain (Bs26D) and phytohormones salicylic acid (SA), abscisic acid (ABA), and ethephone (ET) on the relative expression miRNAs miR156 (**A**), miR159 (**B**), miR160 (**C**), and miR166a (**D**) in wheat plants infested with bird cherry-oat aphid *R. padi*. The samples are indicated as follows: Control—non-bacterized plants and unpopulated with aphids; Bs26D—plants treated with the *B. subtilis* 26D strain before sowing; *R. padi*—non-bacterized and untreated plants populated with aphids; SA—plants treated with salicylic acid; ET—plants treated with ethephone; ABA—plants treated with abscisic acid. Figures present means ± SE (*n* = 6). Columns of each histogram marked with different letters represent the mean values that are statistically different from each other according to Duncan’s test (*p* ≤ 0.05).

**Figure 10:**
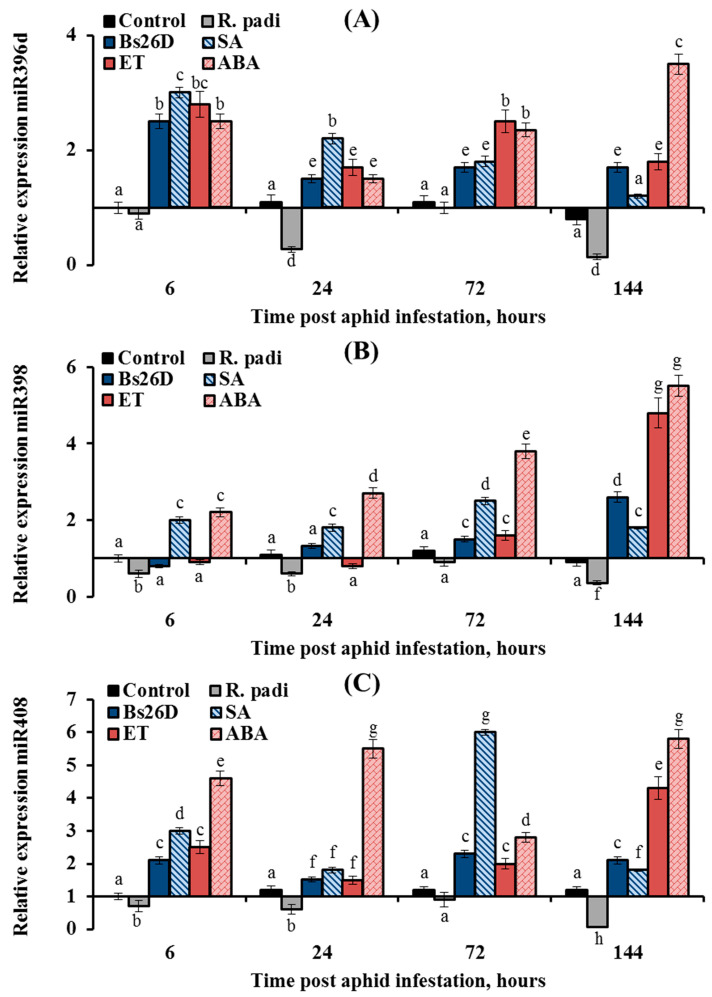
Influence of the *B. subtilis* 26D strain (Bs26D) and phytohormones salicylic acid (SA), abscisic acid (ABA), and ethephone (ET) on the relative expression miRNAs miR396d (**A**), miR398 (**B**), and miR408 (**C**) in wheat plants infested with bird cherry-oat aphid *R. padi*. The samples are indicated as follows: Control—non-bacterized plants and unpopulated with aphids; Bs26D—plants treated with the *B. subtilis* 26D strain before sowing; *R. padi*—non-bacterized and untreated plants populated with aphids; SA—plants treated with salicylic acid; ET—plants treated with ethephone; ABA—plants treated with abscisic acid. Figures present means ± SE (*n* = 6). Columns of each histogram marked with different letters represent the mean values that are statistically different from each other according to Duncan’s test (*p* ≤ 0.05).

In this work, the expression of nine conserved microRNAs (miR156, miR159, miR160, miR164, miR166a, miR393, miR396d, miR398, and miR408) was studied in plants treated with the *B. subtilis* 26D strain and various phytohormones (ABA, SA, and ethylene) during the colonization of plants bird cherry-oat aphid *R. padi* (Figure 8, Figure 9 and Figure 10). Colonization of plants by bird cherry-oat aphids induced the expression of five miRNAs, miR164 (Figure 8B) and miR156, miR159, miR160, and miR166a (Figure 9); the greatest increase in the expression of these miRNAs was found after 6 and 24 h feeding of aphids on plants (Figure 8 and Figure 9). Additionally, colonization of plants by bird cherry-oat aphids inhibited the expression of four miRNAs, miR393 (Figure 8A), miR396d, miR398, and miR408 (Figure 10), throughout the experiment.

The expression of miR393 decreased by 3 and 14 times in wheat plants at 24 and 144 h after *R. padi* colonization compared to the control (Figure 8A). The pre-sowing treatment of wheat seeds with the *B. subtilis* 26D strain supported the transcript level of miR393 near the control values throughout the experiment (Figure 8A). Treatment of plants with ABA acted on the expression of miR393 in a similar manner to the *B. subtilis* 26D strain (Figure 8A). Treatment of plants with SA and ET increased the transcript level of the miR393 24 and 72 h after aphid colonization and then significantly decreased by 5 and 2 times, respectively, compared to the control 144 h after infestation with *R. padi* (Figure 8A).

The expression of miR164 increased by 3.5 times in wheat plants compared to the control 6 h after *R. padi* colonization (Figure 8B). The pre-sowing treatment of wheat seeds with the *B. subtilis* 26D strain led to suppression of miR164 expression by 2–3 times compared to the control throughout the experiment (Figure 8B). Treatment of plants with ET decreased the expression of miR164 by 2–3 times compared to the control throughout the experiment; in other words, ET acted in a similar manner to the *B. subtilis* 26D strain (Figure 8B). 

Treatment of plants with SA supported the expression of miR164 near the control values at the initial stage of *R. padi* feeding and significantly decreased the expression of miR164 by 6.7 times compared to the control 144 h after infestation with *R. padi* (Figure 8B). Treatment of plants with ABA increased the transcript level of the miR164 by 2–4 times compared to the control throughout the experiment (Figure 8B).

The expression of miR156, miR159, miR160, and miR166a increased by 2–6 times in non-bacterized wheat plants and decreased by 1.5–4.4 times in plants treated with the *B. subtilis* 26D strain at the initial stage of feeding aphids after 6, 24, and 72 h of plant colonization by *R. padi* (Figure 9). However, the expression of these miRNAs increased in bacterized wheat plants by 2–3 times compared to the control 144 h after aphid colonization (Figure 9). The effect of SA, ET, and ABA treatment on the expression of miR156, miR159, and miR166a was similar to the effect of the *B. subtilis* 26D strain on this parameter (Figure 9). Treatment of plants with ET decreased the expression of miR160 at 6, 24, and 72 h after infestation by *R. padi* and increased the expression of miR160 by 2.2 times compared to the control 144 h after aphid colonization; in other words, ET acted in a similar manner to the *B. subtilis* 26D strain (Figure 9C). Treatment of plants with SA increased expression of miR160 throughout the experiment in aphid-infested plants (Figure 9C). Treatment of plants with ABA increased the expression of miR160 72 and 144 h after aphid colonization (Figure 9C).

The expression of the three miRNAs, miR396d, miR398, and miR408, was suppressed by the aphid *R. padi*, but it was induced in the *B. subtilis* 26D-treated and aphid-infested plants (Figure 10). The expression of miR396d decreased by 3.7 and 7 times in wheat plants at 24 and 144 h after *R. padi* colonization compared to the control (Figure 10A). The treatment of wheat seeds with the *B. subtilis* 26D strain had a stronger effect on the expression miR396d at the initial stage of feeding aphids after 6 h of plant colonization; the transcript level of miR396d increased by 2.5 times compared to the control (Figure 10A). The effect of SA, ET, and ABA treatment on the expression of miR396d was similar to the effect of the *B. subtilis* 26D strain on this parameter (Figure 10A). However, the effect of hormonal treatment was stronger than bacterial treatment, especially in the later period of feeding aphids after 24, 72, and 144 h of plant colonization (Figure 10A). The expression of miR398 decreased by 2–3 times in wheat plants after *R. padi* colonization compared to the control throughout the experiment (Figure 10B). The treatment of wheat seeds with the *B. subtilis* 26D strain had a stronger effect on the expression miR398 at the later stage of feeding aphids after 144 h of plant colonization; the transcript level of miR398 increased by 2.6 times compared to the control (Figure 10B). The effect of SA, ET, and ABA treatment on the expression of miR398 was similar to the effect of the *B. subtilis* 26D strain on this parameter (Figure 10B). The expression of miR408 decreased by 16.7 times in wheat plants at 144 h after *R. padi* colonization compared to the control (Figure 10C). The treatment of wheat seeds with the *B. subtilis* 26D strain increased the transcript level of the miR408 by 2–6 times compared to the control throughout the experiment (Figure 10C). The strongest effect on the expression of miR408 was treatment by SA and ABA (Figure 10C).

Thus, our results showed that the *B. subtilis* 26D strain, SA, ABA, and ethylene regulated the expression of nine conservative miRNAs in a similar manner.

## 4. Discussion

PGPB, especially endophytes, are important biocontrol agents for a wide range of different pests [8,9,55]. PGPB, as inducers of ISR via the priming mechanism, have been attracting considerable attention from scientists worldwide [56,57]. Priming induced by bacteria provides faster and longer-term plant protection throughout the growing season with low physiological costs [56,57]. The mechanisms of priming have not been fully disclosed, but it has been established that during the development of PGPB-mediated ISR, ROS are generated, hormonal signaling pathways are activated, and PR proteins are accumulated [58,59]. Recently, sRNAs have been considered important regulators of plant protection against pathogens and pests [21,36]. This work is focused on studying the role of the endophytic bacteria *B. subtilis* 26D in the development of defense reactions along the priming pathway in wheat plants against the bird cherry-oat aphid *R. padi*.

*B. subtilis* 26D is a growth-promoting endophytic bacteria that triggers ISR in wheat against various pathogens and pests [12,44,60]. Our early work showed that *B. subtilis* 26D had an indirect effect on the viability of Greenbug aphid *S. graminum* and the endurance of wheat plants since it triggered ISR in wheat plants due to the synthesis of lipopeptide surfactin [12]. In this work, the pre-sowing treatment of wheat seeds with the *B. subtilis* 26D strain effectively increased wheat leaf growth during *R. padi* feeding, reduced aphid fecundity, and increased aphid mortality on plants (Table 1), suggesting that *B. subtilis* 26D activates plant immune response. Our early work showed that treatment with *B. subtilis* 26D induced ISR in wheat against *S. graminum* by activating the expression of markers of the SA- and ethylene-dependent *PR* genes, as well as due to the effect on the plant redox metabolism [12].

In this work, treatment with *B. subtilis* 26D induced oxidative burst in wheat plants infested with bird cherry-oat aphid (Figure 3). Oxidative burst is considered a typical reaction to develop resistance to insects feeding on phloem sap, which can lead to direct damage to the pest or induction of defensive reactions and ISR [4,8,11]. It has been reported that the effect of the endophytic bacteria *B. velezensis* YC7010 on the roots of Arabidopsis can induce systemic resistance against aphids through H_2_O_2_ accumulation, cell death, and callose deposition in leaves [61]. In addition, treatment with *B. subtilis* 26D induced peroxidase activity and suppressed the catalase activity in plants infested with bird cherry-oat aphids (Figure 3). It was shown that the activation of apoplastic peroxidases together with a high level of H_2_O_2_ led to the reorganization and strengthening of cell walls due to lignification and the synthesis of phenols [4,8,11]. Low catalase activity in aphid-infected tolerant crop phenotypes contributed to the development of oxidative burst and tolerance [62]. A number of studies have shown that bacteria-treated plants inoculated with insects exhibited increased POD activity, demonstrating the improved strategy for plant defense against insects induced by bacteria [11,47,63,64].

In this work, analysis of gene expression of SA, JA, ethylene, and ABA signaling pathways markers showed that feeding *R. padi* on control plants led to the activation of only genes associated with JA/ethylene-dependent response (*TaLOX*, *TaERF1*, *TaPR6,* and *TaACS*, *TaEIN3*, *TaPR3*) (Figure 4, Figure 5 and Figure 6). In the literature, high basal levels of JA and defense-related lipoxygenases (LOX) are considered an indicator of strong plant colonization by aphids and tolerance to damage caused during aphid feeding [65]. Some studies have shown that the JA signaling pathway was activated in both susceptible and plants resistant to aphids. Induction of the SA signaling pathway was faster and stronger only in resistant genotypes [3]. The role of SA in the protective response to aphid feeding has been noted in many plant species [3]. There is much less information about the role of ethylene and ABA in protecting plants from aphids, and often this data is contradictory. Some studies have observed increased ethylene levels in barley cultivars resistant to aphids *S. graminum* and *R. padi* [15]. In a series of studies on Arabidopsis plants inhabited by *M. persicae* and on wheat plants colonized by English Grain aphid *Sitobion avenae*, it has been shown that activation of the ethylene signaling pathway is necessary for the polymerization of phloem lectin proteins and glucans that prevent aphid feeding [66,67,68,69]. Thus, the accumulation of ABA in soybean plants susceptible to aphids was previously shown [70]. Recently, it has been shown that high levels of ABA and ABA-related gene transcripts accumulate specifically in the tolerant soybean genotype and are apparently necessary for the development of resistance [65].

The treatment with *B. subtilis* 26D induced genes of the SA-, ethylene- and ABA-signaling pathways in wheat plants inhabited by bird cherry-oat aphids; *B. subtilis* 26D influenced at three levels—hormone synthesis, signal transmission (activation of TF genes expression), and gene expression of defensive protein such as *PR1* and *PR3* (Figure 4, Figure 5 and Figure 6). It has been established that during an aphid attack, the PR1 protein is involved in the regulation of callose deposition, which plays an important role in the protective reactions of plants against aphid colonization [71,72]. PR3 (chitinase) from various strains of *Bacillus* spp. caused increased mortality in melon and potato aphids and caterpillars of the tobacco cutworm *Spodoptera litura*, blocking the activity of most gut enzymes that are necessary for feeding insects [73,74]. However, the effect of *B. subtilis* 26D on the JA signaling pathway was insignificant and transient after 24 h feeding of *R. padi* (Figure 4, Figure 5 and Figure 6). It has been shown that various PGPBs are able to induce ISR via SA, JA, ethylene, and ABA signaling pathways against pathogens and pests [14]. Thus, strains of *Bacillus pumilis* and *Bacillus amyloliquefaciens* induced systemic resistance in cotton plants against the bollworm *Spodoptera exigua* via the ethylene/JA-dependent pathway [75]. *Pseudomonas fluorescens* induced systemic resistance in *Arabidopsis thaliana* against the green peach aphid *Myzus persicae* via SA- and JA-dependent pathways [76]. Treatment of bean plants with the *B. amyloliquefaciens* strain FZB42 reduced the reproduction of the pea aphid *Acyrthosiphon pisum* and increased the content of both SA and JA in plants [9]. 

Despite growing interest in bacterial-mediated ISR against sap-sucking insects, the main molecular and chemical mechanisms of this phenomenon remain unclear [7,11,16,61].

Not only do hormonal signaling pathways play an important role in the regulation of bacteria-mediated ISR developing on the priming pathway, the RNA interference system and sRNAs also play a regulatory role [34,77]. DCL and AGO proteins are the most important components of the RNA interference machinery in plant defense since sRNAs are generated by DCL and function through AGO to suppress target genes [21]. In addition, phytohormones are able to regulate all components of the RNAi system [36].

In this work, analysis of gene expression of *AGO* and *DCL* in control aphid-infested wheat plants showed an increase in mRNA content of all six genes (*AGO1*, *AGO2*, *AGO4*, *AGO5*, *DCL2,* and *DCL4*) only 72 and 144 h after the colonization of plants with *R. padi* (Figure 7). Conversely, an increase in the expression of six genes of AGO and DCL in *B. subtilis* 26D-treated and aphid-infested wheat plants was detected after 6 h of feeding of *R. padi*, and the expression of these genes reached maximum values (10–20-fold increase) after 72 h of plant colonization (Figure 7). Thus, the treatment of plants with the bacteria accelerated and enhanced the plants’ defense response, which is consistent with the priming theory [56,57]. Since *B. subtilis* 26D induced SA, ethylene, and ABA signaling pathways in aphid-infested plants, the influence of the phytohormones SA, ethylene, and ABA on the expression of the *AGO* and *DCL* genes was tested (Table 3 and Table 4). Treatment of plants with SA, ethylene, and ABA showed that all hormones affected the viability of aphids and plant endurance, suggesting that hormones activated plant immune response to *R. padi* (Table 2).

Treatment of plants with SA and ET increased expression of the *AGO1* gene after 24 h of aphid feeding, and treatment of plants with ABA increased the mRNA content of the *AGO1* gene only after 72 and 144 h colonization; in other words, SA and ET acted in a similar manner to *B. subtilis* 26D, but to a lesser extent (Figure 7, Table 3). It has been shown that *AGO1* gene expression is induced by both the JA signaling pathway and the SA and ethylene signaling pathways [21,78]. The role of AGO1 in protecting plants against insects is to induce the biosynthesis of glucosinolate, which inhibits the feeding of peach aphids on *A. thaliana* plants [30].

Only ET treatment affected *AGO2* gene expression in a similar manner to *B. subtilis* 26D, but to a lesser extent (Figure 7, Table 3). Previously, the influence of the ethylene signaling pathway on increasing the expression of the *AGO2* gene during the development of resistance to viral infection was shown [79]. The AGO2 protein’s role in enhancing the secretion of the PR1 protein, a marker of the SA signaling pathway, was revealed, which led to an increase in plant resistance to bacterial infection [34].

All phytohormones SA, ethylene, and ABA increased the expression of the *AGO4* gene in aphid-infested wheat plants; they began to act after 24 h of aphid feeding, but only ET had the strongest effect on the transcription of the *AGO4* gene (Table 3). AGO4 proteins are the most studied AGOs in the pathway of RNA-directed DNA methylation (RdDM) in the formation of plant resistance to bacterial pathogens [21]. Recently, it has been shown that in *ago4* mutants with an impaired function of RdDM, the expression of TF of the ethylene signaling pathway from the ERF family, which is involved in the defense response of *A. thaliana* infested by green peach aphid *M. persicae*, was reduced [33]. However, the biological functions of AGO4 are complex and require further study [54]. In this study, bird cherry-oat aphids induced expression of the *AGO4* gene in wheat plants, but treatment with *B. subtilis* 26D had a much stronger effect on the transcription of this gene. This can most likely be associated with the development of resistance against aphids and activation of the ethylene signaling pathway by bacteria (Figure 7). Thus, recently, it has been shown that activation of the ethylene signaling pathway requires methylation of genes that control ethylene synthesis, signaling in the cytoplasm and nucleus, and response to stress factors [80].

All phytohormones SA, ethylene, and ABA increased the expression of the *AGO5* gene in aphid-infested wheat plants; they acted in a similar manner to *B. subtilis* 26D (Figure 7, Table 3). ET and ABA had the strongest effect on the transcription of the *AGO5* gene (Table 3). Recently, it was shown that AGO5 has a critical role in regulating the response against colonization by *Diuraphis noxia* since the knockdown of AGO5 in the resistant wheat line Tugela DN resulted in a fully susceptible phenotype [32].

It is known that the DCL2 and DCL4 proteins, which provide the processing of double-stranded RNA, are involved in the development of plant defense responses against viruses and pathogens [34,81]. The participation of DCL4 in the development of tobacco plants *Nicotiana attenuata* resistance against the larvae of the *Manduca sexta* was shown [82]. The melon aphid *Aphis gossypii* induced the gene expression of all DCL classes in cotton plants, including the *DCL2* and *DCL4* genes. The mRNA content of the *DCL2* gene was 6 times higher in the resistant cultivar than in the susceptible one [31]. In this study, the bird cherry-oat aphid induced the mRNA accumulation of the *DCL2* gene earlier and stronger than the transcripts accumulation of the *DCL4* gene (Figure 7). Treatment with *B. subtilis* 26D induced an increase in transcript levels of both *DCL2* and *DCL4* genes, but transcriptional activation of the *DCL2* gene was detected after 6 h of colonization (Figure 7). This can be associated with the development of plant resistance to *R. padi* [31]. Our results are consistent with recently obtained data on mutants of *A. thaliana dcl1-9* defective in the synthesis of DCL proteins. This work shows that the *Bacillus cereus* AR156 required DCL proteins to induce resistance in plants [83]. As our results showed, all phytohormones SA, ethylene, and ABA increased the expression of the *DCL2* and *DCL4* genes in aphid-infested wheat plants (Table 4). Some studies have shown the sensitivity of DCL2 to SA and ethylene during the development of resistance against viral infection [34,79]. Moreover, sensitive elements to the phytohormones ethylene, gibberellins (GA), and methyl JA were found in the promoter region of the cotton genes *DCL2*, *DCL3,* and *DCL4* [31].

Thus, *B. subtilis* 26D regulated the expression of genes encoding enzymes of the RNA interference system *AGO* and *DCL* through activation of the hormonal signaling pathways SA, ethylene, and ABA. sRNAs are mediators of hormonal crosstalk and coordinate all plant hormonal responses associated with both developmental and defense programs [36]. 

Recent studies have revealed that small RNAs are critical regulators of the plant immune response [21,36]. The first microRNA (miRNA) identified to involve the immune response was miR393, which was induced by flg22 to repress auxin signaling by silencing its receptors [22]. In addition to miR393, other miRNAs, such as miR159, miR160, miR166, miR167, miR396, miR398, and miR408, also play essential roles in plant defense [21,23,24,25,26,27].

In this work, the expression of nine conserved miRNAs (miR156, miR159, miR160, miR164, miR166a, miR393, miR396d, miR398, and miR408) was studied. Colonization of plants by bird cherry-oat aphids induced the expression of four miRNAs, miR156, miR159, miR160, and miR166a, at the initial stage of feeding aphids after 6, 24, and 72 h of plant colonization and the expression of one, miR164, increased throughout the experiment (Figure 8 and Figure 9). Treatment with *B. subtilis* 26D had the opposite effect on the expression of these miRNAs (Figure 8 and Figure 9). Our results suggest that an increase in the transcript level of miR156, miR159, miR160, and miR166a at the initial stage of feeding aphids was associated with plant susceptibility to insect colonization. This assumption is confirmed by previous studies that examined the expression of conserved miRNAs miR156, miR159, miR160, and miR166 during an infestation of rice, Arabidopsis, melon, chrysanthemum, and tobacco by various aphid and whitefly species. [24,26,28,84]. The whitefly *Bemisia tabaci*, the green peach aphid *Myzus persicae*, the melon aphid *Aphis gossypii,* or chrysanthemum aphid *Macrosiphoniella sanbourni* induced the expression of these miRNAs at the initial stage of feeding pest after 3, 6, 12, 24, and 48 h colonization, and the authors of these studies suggested that these miRNAs may regulate hormonal signaling pathways to improve insect feeding [24,26,28,84]. 

Unfortunately, these works did not study the long-term responses of plants to insect colonization, which begins after the fifth day of colonization by phloem-feeding insects. During the period from the 5th to the 10th day of pest infestation, major physiological changes occur in plants, which do not always coincide with the rapid response reaction [4]. Early responses of resistant plants are characterized by increased ROS generation and activation of basal immunity, and then the system of ROS detoxification and growth restoration in plants is triggered by defense systems during long-term responses [4]. In this work, the expression of miRNAs was studied during long-term responses on the sixth day of aphid feeding (Figure 8 and Figure 9). Colonization of plants by *R. padi* inhibited the expression of four miRNAs, miR156, miR159, miR160, and miR166a, on the sixth day of aphid feeding, and the treatment with *B. subtilis* 26D induced the expression of these miRNAs due to activation of SA, ethylene, and ABA signaling pathways (Figure 9). This induction of miR156, miR159, miR160, and miR166a expression may be associated with the development of plant resistance to aphids.

The different patterns of miRNA expression and the opposite effects of *R. padi* and *B. subtilis* 26D could be related to the fact that miRNAs performed different roles in plant response to phloem-feeding insects, which manifested themselves in the influence on hormonal signaling pathways and the synthesis of secondary metabolites. It is known that a high level of miR156 in plants repress SPL9 (squamosa promoter binding protein-like), which leads to activation of the JA pathway [85], which is an indicator of strong colonization of plants by aphids with the formation of extensive damage [3,65]. In addition, miR156 controls the ABA–ethylene–IAA crosstalk [86]. Recent studies have shown that SPL9 physically interacted with Abscisic Acid Insensitive 5 (ABI5), a master transcription factor in ABA signaling, thus promoting its association with the promoters of ABA-responsive genes, which demonstrates the negative role of miR156 in the ABA-dependent response [87]. Our results showed that the transcript level of the *TaABI5* gene was decreased, and miR156 expression was increased in aphid-infested plants (Figure 5 and Figure 9). Alternatively, miR156 promotes the accumulation of anthocyanins [88], which protect plants by influencing the behavior, growth, and development of insects [89]. 

MiR159 suppresses the expression of *GAMYB101* and *GAMYB33* genes by suppressing GA signaling and programmed cell death, which is activated by these TFs [24,29,86]. It is believed that miR159 regulates the GA–ABA–ethylene crosstalk to control processes associated with programmed cell death [86]. It was shown that ABA signaling is activated due to the fact that MYB33 binds to the *ABI5* promoter; on the contrary, ABA signaling is suppressed by miR159 [86,90]. This may be related to *R. padi* susceptibility since ABA induces plant resistance against aphids, as shown by our results (Table 2). Additionally, miR159 can affect peroxidase genes, suppressing their expression [91]. This can lead to the susceptibility of plants to aphids (Figure 3). Alternatively, miR159 induces carotenoid and flavonoid biosynthesis [88], which can provide plants protection from insects.

A recent study uncovered a new role of ABI5 in phase changes of vegetative growth in plants’ juvenile-to-adult transition associated with miR156 and miR159 [90]. Increased levels of ABI5 promote the expression of miR156 to keep plants in the juvenile phase because juvenile plants are capable of a higher degree of plasticity and are more resistant to stresses, and miR159, by reducing the expression of ABI5, allows plants to recover growth after stress and after high concentrations of ABA under adverse conditions [90]. miR159 accumulates in response to exogenous ABA, and ABI3 regulates miR159 accumulation [92]. In our work, ABA treatment increased the expression of miR156 and miR159 after 144 h of plant colonization by aphids, which could increase plant resistance to stress (Figure 9). The *B. subtilis* 26D treatment acted similarly to ABA (Figure 9).

MiR160 suppresses the auxin signal by targeting genes encoding auxin response factors 10 (*StARF10*) and *StARF16* [86]. However, it was shown that in the absence of miR160, AtARF10 increased the expression of ABA-regulated genes. Furthermore, the inhibition of the ABA pathway by miR160 could also promote the expression of miR167, which enhanced JA biosynthesis [86]. It is known that JA signaling is an indicator of strong colonization of plants by aphids with the formation of extensive damage [3,65]. However, recent studies have shown other functions of miR160. For example, miR160 positively regulated callose accumulation, the SA signaling pathway, and activated the expression of the *PR1* marker gene [93]. In addition, rice lines overexpressing miR160 increased the accumulation of H_2_O_2_ in response to a fungal infection [94]. Our results showed that accumulation of H_2_O_2_, expression of the PR1 gene, and activation of the SA pathway are associated with the development of resistance against *R. padi* and were induced by *B. subtilis* 26D (Figure 3, Figure 4, Figure 5 and Figure 6). In addition, the treatment of plants with SA induced the expression of miR160 throughout the experiment (Figure 9).

The miR166 family members (miR166a–miR166g) have several major target genes that encode HD-ZIP III transcription factors [95]. MiR166 suppressed HD-ZIP III gene expression and caused increased phloem formation and decreasing xylem formation [96], which could improve aphid feeding. The decline in miR166 expression in transgenic maize plants led to ABA content induction and IAA content reduction [95]. Thus, miR166 suppressed ABA accumulation and ABA-dependent defense responses, which could be associated with susceptibility to *R. padi* in our study. Furthermore, the ethylene-insensitive 2 (EIN2) gene, a mediator of ethylene-dependent defense responses in plants, was identified as a novel target gene for miR166 [97]. Activation of miR166 suppressed ethylene signaling pathway in rice during fungal infection [97]. Our results showed that ET treatment decreased the expression of miR166 at the initial stage of feeding aphids after 6, 24, and 72 h of plant colonization by *R. padi* (Figure 9). However, targets of miR166 ATHB-8 and ATHB-15 are associated with the regulation of secondary cell wall differentiation and lignification [96], which may be important during insect attacks to strengthen the cell wall and form a protective barrier. This fact may explain the positive effects of B. subtilis 26D, SA, ET, and ABA treatments on the expression of miR166 after 144 h of plant colonization during the recovery phase (Figure 9).

The central role of miR164 is implication in the phytohormone-mediated regulation of leaf senescence [86]. IAA induced miR164 expression to repress the *NAC2* target gene and reduce leaf senescence [86]. The ethylene signaling pathway TF EIN3 binds to the miR164 promoter, reduces its transcript levels, and derepresses *NAC2*; thereby, ethylene promotes progressive leaf senescence, which limits aphid reproduction and promotes plant resistance against insects [24,98]. Our results showed that ET treatment decreased the expression of miR164 throughout the experiment (Figure 8). The *B. subtilis* 26D treatment acted similarly to ET (Figure 8). Recently, the role of miR164-MYB and miR164-NAC modules in stress response regulatory pathways in leaves associated with ABA was suggested [99]. Some MYB and NAC genes could enhance or weaken the ABA signaling pathway, which means miR164 is able to regulate the ABA signaling pathway [99]. In this study, ABA treatment induced miR164 expression throughout the experiment (Figure 8). Perhaps this fact was associated with the regulation of the transcript level of some NACs that negatively regulate plant stress resistance [100]. 

Additionally, colonization of plants by bird cherry-oat aphids inhibited the expression of four miRNAs, miR393, miR396d, miR398, and miR408, throughout the experiment (Figure 8 and Figure 10). Treatment of *B. subtilis* 26D, SA, ET, and ABA increased the expression of these miRNAs (Figure 8 and Figure 10).

The activity of miR393 is associated with suppression of the auxin signaling pathway under biotic and abiotic stress. Thus, miR393 negatively regulated transcripts encoding F-box auxin receptors TIR1, AFB2, and AFB3 and subsequently confers enhanced resistance against *P. syringae* bacteria in Arabidopsis [36]. It has been shown that miR393 is involved in the regulation of ABA–IAA crosstalk under stress [86]. ABA treatment upregulates miR393 biosynthesis, and miR393 represses the perception of the IAA signal [86]. The results of this work showed that SA, ET, and ABA increased the expression of miR393 by 1.5–2 times compared to control, and *B. subtilis* 26D maintained the level of transcripts of this miRNA near control values (Figure 8). The exception was the 144 h recovery phase, where *B. subtilis* 26D, SA, and ET, but not ABA, reduced miR393 expression (Figure 8). This circumstance can be explained by the fact that overexpression of miR393 led to increased synthesis of glucosinolates (GS) and camalexin (CL), and these secondary metabolites are toxic not only to insects but also to a wide range of bacteria [88,89]. 

MiR396 is a negative regulator of mitotic cell division through the downregulation of growth responding factor (GRF) genes in shoot meristems, leaves, and roots and, therefore, controls the balance between growth and immune response [86,101]. It has been shown that miR396 is involved in the control of phytohormone-related genes. Using the triple mutant *grf1/grf2/grf3* and two miRNA-resistant forms, *AtGRF1* and *AtGRF3*, the effect of miR396 on more than 60 genes of six hormonal signaling pathways of ethylene, cytokinins, IAA, GA, JA, and ABA was demonstrated [102]. For example, in the triple mutant grf1/grf2/grf3, the ABA signaling pathway genes *ABA1* and *ABA4* were suppressed [102]. It has also been suggested that miR396 might be an important gateway for both ABA and ET to control cell proliferation in response to biotic and abiotic stresses [86]. In this work, ET and ABA had the greatest effect on the expression of miR396 (Figure 8). Additionally, miR396 is involved in the biosynthesis of carotenoids, flavonoids, and terpenoids [88], which can provide plant protection from insects. 

The activity of two miRNAs, miR398 and miR408, is associated with the redox status of the plant and the synthesis of reactive oxygen species (ROS) [103]. These miRNAs were induced upon treatment with all three phytohormones but to a greater extent upon treatment with ABA and SA (Figure 10). Thus miR398, in *M. oryzae*-infected rice plants increased the activity of superoxide dismutase (SOD), thereby raising the concentration of H_2_O_2_, inducing the expression of PR1 and PR10 defense genes and plant resistance [36,104]. Recently, it has been shown that miR408 in wheat targets catalase genes, an enzyme involved in the degradation of H_2_O_2_ [105]. miR408 is a highly conserved miRNA, which is involved in the regulation of plant growth, development, and stress response. miR408 regulates the growth and development of different plants by downregulating its targets, encoding blue copper (Cu) proteins, and transporting Cu to plastocyanin (PC), which affects photosynthesis and, ultimately, promotes grain yield [27].

Thus, *B. subtilis* 26D regulated the expression of the *AGO* and *DCL* genes, as well as the expression of nine conserved miRNAs (miR156, miR159, miR160, miR164, miR166a, miR393, miR396d, miR398, and miR408) through activation of the hormonal signaling pathways SA, ethylene, and ABA. It was found that *B. subtilis* 26D, SA, ethylene, and ABA had the strongest effect on the expression of the *AGO4*, *AGO5* and *DCL2*, *DCL4* genes. Analysis of the expression pattern of these nine miRNAs revealed positive and negative effects of the same miRNAs on the induction of defense responses at different stages of aphid feeding. Positive and negative effects of *B. subtilis* 26D, SA, ethylene, and ABA on the expression of all nine conserved miRNAs were also found. It is assumed that the different patterns of miRNA expression and the diverse effects of *B. subtilis* 26D and phytohormones could be related to the fact that miRNAs performed multiple roles in plant response to phloem-feeding insects, which manifested themselves in the influence on hormonal signaling pathways, redox metabolism, and the synthesis of secondary metabolites.

## 5. Conclusions

Thus, our results showed that the endophytic bacteria *B. subtilis* 26D induced ISR in wheat plants against bird cherry-oat aphids, increasing the expression of *PR1* and *PR3* genes the most, activating the RNAi mechanism, and regulating the expression of nine conserved miRNAs through activation of the ethylene, SA, and ABA signaling pathways, which was demonstrated using treatments with phytohormones. Treatment of plants with SA, ethylene, and ABA acted in a similar manner to the *B. subtilis* 26D strain on the induction of the expression of the *AGO* and *DCL* genes, as well as the expression of nine conserved miRNAs. 

The protective response of wheat plants against bird cherry-oat aphids was associated with the generation of ROS and changes in the activity of redox enzymes, as well as with the synthesis of protective proteins, which were regulated by phytohormones. It was found that miR156, miR159, miR164, and miR166a suppressed the ABA signaling pathway, and miR156 and miR159 influenced the expression of the TF of the ABA signaling pathway TaABI5. In addition, miR166 could suppress the ethylene signaling pathway. MiR160, miR398, and miR408 were most likely involved in the induction of oxidative burst. In addition, miR160 and miR398 could activate the PR1 protein and SA signaling pathway. It is known that miR156, miR159, miR393, and miR396 can influence the synthesis of secondary metabolites of flavonoids, terpenoids, and glucosinolates, which are toxic to many insects.

This work was the first to study the influence of the endophytic bacteria *B. subtilis* 26D on RNA interference components and microRNA expression of wheat during bird cherry-oat aphid colonization. Our study provides new data to further elucidate the fine mechanisms of bacterial-induced priming. However, further extensive work is needed to fully unravel these mechanisms.

## Figures and Tables

**Table 1:** The influence of *B. subtilis* 26D strain on the viability indicators of the bird cherry-oat aphid and the endurance indicators of wheat plants infested with *R. padi*.

Indicators of Different Types of Resistance against Aphids		Variant of Treatment	
		Water	*B. subtilis* 26D
Aphid Viability Indicators (Antibiosis)	Fecundity, (nymphs/Seedling)	57.9 ± 3.9 a	38.6 ± 2.2 b
	Mortality,%	9.5 ± 1.7 a	41.4 ± 2.2 b
	Propagation Coefficient (PC)	2.9 ± 0.3 a	1.3 ± 0.1 b
Plants Endurance	Growth Rate of the 1st Leaf, % of Control *	76.7 ± 3.2 a	101.7 ± 5.1 b
	Growth Rate of the 2nd Leaf, % of Control *	80.3 ± 3.9 a	106.4 ± 3.9 b

* Growth rate of the 1st or 2nd leaf of control, non-treated with bacteria and non-populated with aphids is 100%. The variants in the one line marked with different letters represent the mean values that are statistically different from each other according to Duncan’s test (*n* = 15, *p* ≤ 0.05).

**Table 2:** The influence of phytohormones on the viability indicators of the bird cherry-oat aphid and the endurance indicators of wheat plants infested with *R. padi*.

Indicators of Different Types of Resistance against Aphids		Variant of Treatment			
		Water	SA	ET	ABA
Aphid Viability Indicators (Antibiosis)	Fecundity (nymphs/Seedling)	57.9 ± 3.9 a	36.5 ± 2.2 b	34.5 ± 1.4 b	25.3 ± 1.7 c
	Mortality,%	9.5 ± 1.7 a	45.2 ± 2.7 b	47.8 ± 3.1 b	54.8 ± 4.4 c
	Propagation Coefficient (PC)	2.9 ± 0.3 a	1.3 ± 0.1 b	1.3 ± 0.1 b	0.9 ± 0.1 c
Plant Endurance	Growth Rate of the 1st Leaf, % of Control *	76.7 ± 3.2 a	98.8 ± 4.9 b	97.3 ± 5.2 b	85.0 ± 4.3 c
	Growth Rate of the 2nd Leaf, % of Control *	80.3 ± 3.9 a	96.6 ± 4.7 b	92.8 ± 4.1 b	82.0 ± 4.5 a

* Growth rate of the 1st or 2nd leaf of control, non-treated with phytohormones and non-populated with aphids, is 100%. The variants in the one line marked with different letters represent the mean values that are statistically different from each other according to Duncan’s test (*n* = 15, *p ≤* 0.05).

**Table 3:** Influence of phytohormones on the relative expression of genes encoding RNAi enzymes *AGO1*, *AGO2*, *AGO4,* and *AGO5* in wheat plants infested with bird cherry-oat aphid *R. padi*.

Gene	Treatments	Time Post Aphid Infestation, Hours			
		6	24	72	144
*AGO1*	Control	1.0 ± 0.1 a	1.0 ± 0.1 a	0.9 ± 0.1 a	1.1 ± 0.1 a
	*R. padi*	0.9 ± 0.1 a	0.9 ± 0.1 a	1.4 ± 0.2 ab	1.6 ± 0.1 b
	SA	1.3 ± 0.1 ab	1.0 ± 0.1 a	1.4 ± 0.1 ab	1.2 ± 0.1 a
	SA + *R. padi*	1.0 ± 0.1 a	1.7 ± 0.2 b	1.7 ± 0.1 b	1.2 ± 0.1 a
	ET	0.8 ± 0.1 a	1.0 ± 0.1 a	1.2 ± 0.1 a	1.5 ± 0.1 b
	ET + *R. padi*	1.0 ± 0.1 a	1.5 ± 0.1 b	1.4 ± 0.1 ab	1.3 ± 0.2 ab
	ABA	1.2 ± 0.1 a	1.3 ± 0.1 ab	0.7 ± 0.1 c	1.0 ± 0.1 a
	ABA + *R. padi*	1.1 ± 0.1 a	0.9 ± 0.1 a	1.3 ± 0.2 ab	4.0 ± 0.6 c
*AGO2*	Water	1.0 ± 0.1 a	1.0 ± 0.1 a	1.1 ± 0.1 a	1.1 ± 0.1 a
	*R. padi*	1.2 ± 0.1 a	1.0 ± 0.1 a	1.3 ± 0.1 ab	1.9 ± 0.2 b
	SA	1.1 ± 0.1 a	1.0 ± 0.1 a	1.3 ± 0.2 ab	1.5 ± 0.1 b
	SA + *R. padi*	0.9 ± 0.1 a	0.9 ± 0.1 a	1.5 ± 0.2 b	1.2 ± 0.1 a
	ET	0.8 ± 0.1 a	1.3 ± 0.2 ab	1.0 ± 0.1 a	1.4 ± 0.1 ab
	ET + *R. padi*	1.0 ± 0.1 a	1.5 ± 0.1 b	2.1 ± 0.2 b	1.8 ± 0.3 b
	ABA	1.0 ± 0.1 a	1.5 ± 0.1 b	1.6 ± 0.1 b	1.2 ± 0.1 a
	ABA + *R. padi*	1.0 ± 0.1 a	1.0 ± 0.1 a	2.4 ± 0.2 be	2.8 ± 0.6 d
*AGO4*	Water	1.0 ± 0.1 a	1.0 ± 0.1 a	1.0 ± 0.1 a	0.9 ± 0.1 a
	*R. padi*	1.2 ± 0.1 a	1.4 ± 0.1 ab	5.0 ± 0.9 d	1.9 ± 0.3 b
	SA	1.5 ± 0.1 b	2.6 ± 0.4 c	2.4 ± 0.1 be	3.7 ± 0.5 c
	SA + *R. padi*	1.0 ± 0.1 a	4.5 ± 0.3 d	5.5 ± 1.0 d	6.7 ± 0.3 e
	ET	0.8 ± 0.1 a	1.2 ± 0.2 a	2.8 ± 0.3 e	1.2 ± 0.5 a
	ET + *R. padi*	1.8 ± 0.2 b	6.7 ± 0.5 e	7.6 ± 0.9 f	10.9 ± 0.8 f
	ABA	1.3 ± 0.1 ab	1.6 ± 0.3 b	0.7 ± 0.1 c	1.7 ± 0.1 b
	ABA + *R. padi*	1.1 ± 0.1 a	2.2 ± 0.1 c	2.0 ± 0.2 b	3.9 ± 0.6 c
*AGO5*	Water	1.0 ± 0.1 a	1.0 ± 0.1 a	0.9 ± 0.1 a	1.2 ± 0.1 a
	*R. padi*	0.8 ± 0.1 a	0.3 ± 0.1 f	2.3 ± 0.4 be	1.8 ± 0.1 b
	SA	1.3 ± 0.1 ab	1.5 ± 0.2 b	2.3 ± 0.1 be	2.9 ± 0.1 d
	SA + *R. padi*	1.3 ± 0.1 ab	1.5 ± 0.1 b	2.6 ± 0.2 e	2.0 ± 0.1 b
	ET	0.9 ± 0.1 a	0.6 ± 0.2 f	2.2 ± 0.1 be	1.2 ± 0.2 a
	ET + *R. padi*	1.2 ± 0.1 a	2.6 ± 0.5 c	2.6 ± 0.1 e	13.2 ± 0.3 g
	ABA	1.3 ± 0.1 ab	1.4 ± 0.2 ab	1.5 ± 0.1 b	1.6 ± 0.1 b
	ABA + *R. padi*	1.2 ± 0.1 a	1.5 ± 0.1 b	3.0 ± 0.1 e	9.7 ± 0.8 f

The samples are indicated as follows: Control—untreated plants and unpopulated with aphids; *R. padi*—plants populated with aphids; SA—plants treated with salicylic acid; ET—plants treated with ethephone; ABA—plants treated with abscisic acid. Expression values were normalized to the housekeeping gene *TaRLI* as an internal reference and expressed relative to the normalized expression levels in control plants. The variants in the same column marked with different letters represent the mean values that are statistically different from each other according to Duncan’s test (*n* = 6, *p* ≤ 0.05).

**Table 4:** Influence of phytohormones on the relative expression of genes encoding RNAi enzymes *DCL2* and *DCL4* in wheat plants infested with bird cherry-oat aphid *R. padi*.

Gene	Treatments	Time Post Aphid Infestation, Hours			
		6	24	72	144
*DCL2*	Control	1.0 ± 0.1 a	1.0 ± 0.1 a	1.0 ± 0.1 a	1.0 ± 0.1 a
	*R. padi*	1.2 ± 0.1 a	0.6 ± 0.1 b	4.3 ± 0.2 b	1.5 ± 0.1 b
	SA	1.2 ± 0.1 a	1.8 ± 0.1 c	0.5 ± 0.1 c	3.1 ± 0.2 c
	SA + *R. padi*	0.9 ± 0.1 a	1.8 ± 0.1 c	5.5 ± 0.1 d	4.0 ± 0.3 d
	ET	1.1 ± 0.1 a	2.2 ± 0.3 c	1.7 ± 0.1 e	2.7 ± 0.1 c
	ET + *R. padi*	1.3 ± 0.1 ab	2.9 ± 0.1 d	1.7 ± 0.2 e	5.0 ± 0.4 e
	ABA	1.0 ± 0.1 a	1.6 ± 0.1 c	0.7 ± 0.1 c	0.2 ± 0.03 f
	ABA + *R. padi*	1.2 ± 0.1 a	0.9 ± 0.1 a	5.6 ± 0.1 d	4.1 ± 0.3 d
*DCL4*	Water	1.0 ± 0.1 a	1.0 ± 0.1 a	1.0 ± 0.1 a	1.0 ± 0.1 a
	*R. padi*	0.9 ± 0.1 a	1.2 ± 0.1 a	1.3± 0.2 ae	3.3 ± 0.2 c
	SA	1.5 ± 0.1 b	1.4 ± 0.1 ac	1.5 ± 0.4 e	1.1 ± 0.1 a
	SA + *R. padi*	1.0 ± 0.1 a	1.3 ± 0.1 ac	1.8 ± 0.1 e	4.9 ± 0.3 e
	ET	0.8 ± 0.1 a	1.3 ± 0.2 ac	1.1 ± 0.1 a	1.7 ± 0.1 b
	ET + *R. padi*	1.8 ± 0.2 b	2.4 ± 0.1 c	1.8 ± 0.2 e	2.0 ± 0.4 b
	ABA	1.3 ± 0.1 ab	0.9 ± 0.1 a	1.0 ± 0.1 a	0.8 ± 0.1 a
	ABA + *R. padi*	1.1 ± 0.1 a	0.8 ± 0.1 a	1.2 ± 0.1 a	6.6 ± 0.3 g

The samples are indicated as follows: Control—untreated plants and unpopulated with aphids; R. padi—plants populated with aphids; SA—plants treated with salicylic acid; ET—plants treated with ethephone; ABA—plants treated with abscisic acid. Expression values were normalized to the housekeeping gene *TaRLI* as an internal reference and expressed relative to the normalized expression levels in control plants. The variants in the same column marked with different letters represent the mean values that are statistically different from each other according to Duncan’s test (*n* = 6, *p* ≤ 0.05).

## Data Availability

Data are contained within the article and supplementary files.

## Supplementary Materials

The following supporting information can be downloaded at https://www.mdpi.com/article/10.3390/microorganisms11122983/s1, Table S1: Primers used for qPCR.; Table S2: Primers used for qPCR; Table S3: Primers used for qPCR.
