# Supplementary material for: Bacillus subtilis 26D Triggers Induced Systemic Resistance against Rhopalosiphum padi L. by Regulating the Expression of Genes AGO, DCL and microRNA in Bread Spring Wheat
Source: Microorganisms. 2023 Dec 14;11(12):2983. doi: 10.3390/microorganisms11122983 (PMC10745712; doi:10.3390/microorganisms11122983)
Supplement: Supplementary file 1 [file microorganisms-11-02983-s001.zip › Supplement-Rumyantsev-06112023.pdf]

# *B. subtilis* 26D Triggers Induced Systemic Resistance against *Rhopalosiphum padi* L. by Regulating the Expression of Genes *AGO*, *DCL* and microRNA in Bread Spring Wheat

**Sergey D. Rumyantsev, Svetlana V. Veselova*, Guzel F. Burkhanova, Valentin Y. Alekseev, Igor V. Maksimov**

Institute of Biochemistry and Genetics, Ufa Federal Research Centre, Russian Academy of Sciences, Prospekt Oktyabrya, 71, 450054 Ufa, Russia; rumyantsev-serg@mail.ru (S.D.R.); guzel_mur@mail.ru (G.F.B.); valentin-1994@yandexx.ru (V.Y.A.); igor.mak2011@yandex.ru (I.V.M.).

* Correspondence: veselova75@rambler.ru; Tel.; +7(917)3423941

**Supplementary Table S1.** Primers used for qPCR

| Genes | GenBank Accession number | Sequence (5'-3') | |
|---|---|---|---|
| | | Forward Primers | Reverse Primer |
| *TaRLI* | AY059462 | TTGAGCAACTCATGGACCAG | GCTTTCCAAGGCACAAACAT |
| *TaLOX* | AK333416 | CGAGAAGATGGTGATGGAGATG | GACACAGCCAAGAGACTAGAA |
| *TaACS1* | U35779 | GAGGTTGTGAACGAGCTAGAAG | CTCGAAACCCTGCAAGAGATAG |
| *TaPR1* | KF196278 | ATAACCTCGGCGTCTTCATC | GCTTATTACGGCATTCCTTTT |
| *TaPR2* | DQ090946 | GCGTGAAGGTGGTGATTT | GTGCCCGTTACACTTGGAT |
| *TaPR3* | AB029936 | ACCTCCTTGGCGTCAGCT | TCGCACCATTATTCCCTT |
| *TaPR6* | EU293132 | GGGCCCTGCAAGAAGTACTG | ACACGCATAGGCACGATGAC |
| *TaICS* | NR175979 | TTCTTAGAGCGTGTGGCATAG | CAGTGTGAGGTTGGCCTATT |
| *TaNCED* | KP099105 | CCTGCTGCCTCTTCTGCT | ACCAAGTGCTCTTCCGTCTC |
| *TaWRKY13* | EF397614 | GAGGAGGAACATGAGCAGAAG | GAACCACCCGAAGTCGAAG |
| *TaPIE1* | EF583940 | ACACATGCCGACGAATCTAC | CCGAGTTCAAACCCAAATTCAC |
| *TaABI5* | KX002271 | CGTGAAGTTCTCTGAGGAAGAAG | GTCAGCGCGAAGATGGAATA |
| *TaEIL1* | KU03083 | GGCTCAACAACCTGGATTTT | CCCTGTCCAAAGAAACCCT |

**Supplementary Table S2.** Primers used for qPCR

| Genes | GenBank Accession number | Sequence (5'-3') | |
|---|---|---|---|
| | | Forward Primers | Reverse Primer |
| *TaRLI* | AY059462 | TTGAGCAACTCATGGACCAG | GCTTTCCAAGGCACAAACAT |
| *TaAGO1* | JQ805149 | ATCAGCCTCCAGTTACCTTTG | GCAGTATGTTCCCGCTTCTAT |
| *TaAGO2* | KY794780 | CTTTCATCGGCTGTTGCTTATG | GAACGAACTCTGACACGGAATA |
| *TaAGO4* | JQ805150 | CTCCAGGCACTGTTGTAGATAAT | AGTGTGTTGGCCTTGTAGTC |
| *TaAGO5* | AK447406 | GAGTCACCATCCTGGAACTATG | GTAACCTCTCCAGCACTCTAATC |
| *TaDCL2* | KY794782 | CGAATCGTCGAACTGGAGAAT | ATCCTCGTAGTGGCAAATGG |
| *TaDCL4* | KY794783 | CTTCACTGGGTGGGAATAGATG | GGTGTGGTTCGTGAGAGATAAA |

**Supplementary Table S3.** Primers used for qPCR

| Genes | GenBank Accession number | Sequence (5'-3') |
|---|---|---|
| RTQAdp | | CTCTGACGAGAACTTGACTTCACTATGACT |
| Ta5S-rRNA | XR_006485435 | GGATGCGATCATACCAGCACT |
| Ta miR156 | MF540049 | TGACAGAAGAGAGTGAGCACA |
| Ta miR159b | MF540051 | TTTGGATTGAAGGGAGCTCTG |
| Ta miR160 | MF540052 | TGCCAGGCTCCCTGTATGCCA |
| Ta miR164 | MF540053 | TGGAGAAGCAGGGCACGTGCA |
| Ta miR166a | AK454189 | TCGGACCAGGCTTCATTCC |
| Ta miR393 | XM_044543780 | TTCCGAAAGGGATCGCATTG |
| Ta miR396d | XM_044476889 | TCCACTGGCTTTCTTGAACTG |
| Ta miR398 | MF540067 | GCTAGTGTTCTCAGGTCGCC |
| Ta miR408 | MF540069 | CTGCACTGCCTCTTCCCTGGC |
